# Pollination effectiveness affects the level of generalisation of a plant species with phenotypically plastic flowers

**DOI:** 10.1093/aobpla/plae065

**Published:** 2025-01-10

**Authors:** José M Gómez, Luis Navarro, Adela González-Megías, Cristina Armas, Francisco Perfectti, Ángel Caravantes, Raquel Sánchez

**Affiliations:** Department of Functional and Evolutionary Ecology, Estación Experimental de Zonas Áridas (EEZA- CSIC), Carretera de Sacramento s/n, E-04120, Almería, Spain; Research Unit Modeling Nature, Universidad de Granada, E-18071, Granada, Spain; Universidade de Vigo, Departamento de Biología Vegetal y Ciencias del Suelo, E-36310, Vigo, Spain; Research Unit Modeling Nature, Universidad de Granada, E-18071, Granada, Spain; Departamento de Zoología, Universidad de Granada, Avda Fuentenueva s/n, E-18071, Granada, Spain; Department of Functional and Evolutionary Ecology, Estación Experimental de Zonas Áridas (EEZA- CSIC), Carretera de Sacramento s/n, E-04120, Almería, Spain; Research Unit Modeling Nature, Universidad de Granada, E-18071, Granada, Spain; Departamento de Genética, Universidad de Granada, Avda Fuentenueva s/n, E-18071, Granada, Spain; Departamento de Zoología, Universidad de Granada, Avda Fuentenueva s/n, E-18071, Granada, Spain; Department of Functional and Evolutionary Ecology, Estación Experimental de Zonas Áridas (EEZA- CSIC), Carretera de Sacramento s/n, E-04120, Almería, Spain

**Keywords:** Brassicaceae, floral phenotypic plasticity, *Moricandia arvensis*, pollination effectiveness, pollination generalisation, pollination specialisation, pollinator per-visit efficiency, pollinator preference

## Abstract

The pollination specialisation/generalisation continuum is a basic concept in pollination biology, usually defined as the number of species or functional groups of pollinators visiting a plant species. The level of specialisation can be affected by the relative abundance of pollinators on flowers, the among-pollinator variation in per-visit efficiency and the intra-population variation in floral traits. Here, we explore how these components influence the degree of generalisation of a Mediterranean herb, *Moricandia arvensis* (Brassicaceae). *Moricandia arvensis* shows a remarkable intra-individual floral polyphenism, with large cross-shaped lilac flowers during spring and small rounded white flowers during summer. We quantified the pollinator assemblages, grouped into functional groups, of six plant populations from SE Spain during spring and summer, experimentally tested their preference for spring and summer floral morphs, their per-visit efficiency, and their effectiveness during each flowering period, estimated as their contribution to plant’s fitness. The level of generalisation differed between floral morphs: spring flowers are visited mainly by large long-tongued bees, while summer flowers are visited by a diverse set including small short-tongued bees, large long-tongued bees, large butterflies and beeflies. The functional groups of pollinators differed in their preferences for floral morphs, with large long-tongued bees, small short-tongued bees and beeflies preferring spring floral morphs, whereas flies and butterflies preferred summer floral morphs. Pollinator per-visit efficiency also varied among functional groups. Bees and butterflies produced many seeds per visit, while beetles, hoverflies and flies produced few or no seeds per visit. By combining floral visits with per-visit efficiency (effectiveness), the spring pollinator assemblage became significantly more specialised and the summer pollinator assemblage significantly more generalised. Our study suggests that, although traditionally neglected in pollination studies, examining pollinator effectiveness is crucial to accurately characterise generalisation–specialisation gradients, rigorously categorising pollination niches and correctly describing the architecture of plant-pollinator interactions.

## Introduction

The relationship between plants and pollinators has been the subject of study by ecologists for more than a century, but interest in understanding the workings of this fundamental ecological interaction has not waned. The study of pollination ranges from detailed mechanistic and experimental studies of specific pairs of plants and floral visitors to pattern-oriented studies of the structure and dynamics of entire plant-pollinator communities and networks. Much progress has been made in understanding interaction from these two perspectives, and our knowledge on the ecology, evolution and coevolution of individual pairwise interactions (Thompson 2005; Willmer 2011; Patiny 2012) or on the architecture and topology of pollination networks is immense (Bascompte and Jordano 2014).

The idea of the specialisation/generalisation continuum in pollination systems connects these two scales of research, a concept that, despite its importance, is not without controversy (Brosi 2016; Armbruster 2017). In fact, there is still no universal consensus on how to define it or how to measure it, nor on what its real consequences are for the ecology and evolution of plant-pollinator interactions (Waser and Ollerton 2006; Armbruster 2017; Phillips *et al.* 2020). Traditionally, pollination specialisation–generalisation has been assessed as the number of animal species or functional groups visiting the flowers of a given plant species in a given locality (Faegri and van der Pijl 1979; Waser and Ollerton 2006; Ollerton *et al*. 2007). This approach ignores the fact that the frequency of visitation varies greatly between different species or groups of floral visitors, and a marked difference in evenness can lead to systems with the same number of species varying in their degree of generalisation (Gómez and Zamora 2006). This limitation is overcome by adopting a niche pollination approach, where pollinators are considered as resources that vary in abundance along resource axes (Johnson 2010; Gómez *et al*. 2015; Phillips *et al*. 2020) and the position of any plant species along the specialisation–generalisation continuum is inferred by the alpha diversity of its pollinator assemblage (Gómez *et al*. 2015).

Most studies exploring the specialisation/generalisation level of pollination systems do not include information on the efficiency as pollinators of those animals visiting the flowers (Armbruster 2017). Not considering pollination efficiency assumes that all floral visitors contribute equally per visit to the reproduction of the plant. However, this is only sometimes true. Many experimental studies have demonstrated that floral visitors differ in their ability to remove and deposit pollen (Rader *et al*. 2012; Page *et al.* 2021), in the proportion of visits in which they act as mere pollen or nectar robbers (Maloof and Inouye 2000; Irwin *et al.* 2010), in the amount of heterospecific pollen they transfer (Moreira-Hernández and Muchhala 2019), in their capacity of fertilising ovules and produce seeds (Motten *et al.* 1981; McGuire and Armbruster 1991; Rader *et al*. 2012; Page *et al*. 2021), and in the genetic diversity of the seeds produced (Valverde *et al*. 2019). Ignoring this reality may distort our estimation of the specialisation–generalisation degree of any pollination system. In fact, the generalisation degree of the pollination system of some plant species changes substantially when this information is considered (Sahli and Conner 2007; Armbruster 2017). The development of experimental and analytical techniques that incorporate the quality of floral visitors as pollinators (Schupp *et al.* 2010, 2017) may help to gain a more accurate understanding of how generalist or specialist a given pollination system is.

Another factor influencing the level of generalisation of a pollination system is related to the existence of intra-population variation in floral traits, especially when the expression of floral traits affects the preference and per-visit efficiency of particular pollinators (Herrera 1995, 2000; Nocentini *et al*. 2013). When this happens, there is a possibility of individual-level variation in the use of resources and the specialisation level (Bolnick *et al*. 2002, 2003). Among the several sources of floral variation, a special type occurs as a consequence of the existence of phenotypic plasticity, the ability of a genotype to produce alternative phenotypes when exposed to different environments (Schlichting and Pigliucci 1998). Phenotypic plasticity is presumed to evolve in response to contrasting selection pressures that arise when organisms confront environmental heterogeneity (Bradshaw 1965; Scheiner 2013; Murren *et al.* 2015). When heterogeneity happens at fine-grained scales, several plastic phenotypes might coexist within the same population (Méndez-Vigo *et al*. 2013; Ramírez *et al*. 2015), each potentially attracting a different set of pollinators.

In this study, we explore how intra-individual phenotypic plasticity and between-pollinator differences in effectiveness may affect the level of specialisation/generalisation in the pollination system of *Moricandia arvensis* (Brassicaceae). This mustard species is particularly appropriate to test this idea because it is visited by a vast and contrasting assemblage of insects (Gómez *et al.* 2022 a) and exhibits a remarkable intra-individual phenotypic plasticity in the form of extreme floral polyphenism (Gómez *et al.* 2020, 2024). The existing terminology to estimate the functional role of floral visitors as pollinators is diverse. Here, we studied pollination effectiveness using a framework recently broadened to all mutualistic interactions (Schupp *et al.* 2010, 2017). In this framework, effectiveness is ideally viewed as the contribution of one pollinator to the plant’s fitness and is decomposed into a quantity component (QTC) and a quality component (QLC). The QTC is the number of interaction events between a given pollinator species or functional group and a plant species, and it is ideally measured as the number of pollen grains deposited by that pollinator or, when this is not available, the number of floral visits made to the plant species. This is because, strictly speaking, a pollination event -an interaction in which the plant receives a service from the pollinator- is the transfer of a pollen grain from the anther to the stigma and not the simple visit of a floral visitor. QTC includes the performance of pollinators in terms of pollen transferred or deposited onto stigmas (Primack and Silander 1975; Ne’eman *et al.* 2010) or, sometimes, pollen removal (Inouye *et al.* 1994; Sahli and Conner 2007). On the other hand, QLC is a measure of the probability that an interaction event results in the production of seeds and it is measured as the probability that an ovule pollinated by a given pollinator will produce a new reproductive adult or, as a proxy, the number of seeds produced per visit of that pollinator (Schupp *et al.* 2017; Valverde *et al.* 2019). QLC is analogous to those metrics quantifying per-visit or single-visit efficiency or performance of pollinators in terms of seed production (Inouye *et al.* 1994; Olsen 1997; Ne'eman *et al.* 2010; Sahli and Conner 2007; Page *et al.* 2021). Finally, effectiveness is quantified in our framework as QTC × QLC, which is similar to the term pollinator importance (Herrera 1987; Olsen 1997; Sahli and Conner 2006, 2007). Variation in effectiveness among interacting organisms can be visualised in a two-dimensional effectiveness landscape where each species’ location is determined by its values of quantity (x-axis) and quality (y-axis) components (Schupp *et al.* 2017). Some properties of the spatial configuration of the effectiveness landscape can be used to understand functional aspects of the interaction (Gómez *et al.* 2022*b*). The dispersion of effectiveness values across the landscape can inform about the functional redundancy of the different pollinators, whereas the correlation between components can inform on the functional specialisation degree of the system (Gómez 2022*b*). Under this scenario, the goals of this study are (i) to assess the quantity and quality components and the effectiveness of the main pollinator functional groups of *M. arvensis*; (ii) to explore how the generalisation degree of the system changes when considering their effectiveness and (iii) to check whether these patterns are influenced by the within-individual floral plasticity exhibited by the plant.

## Methods

### The study species

**Figure 1.**
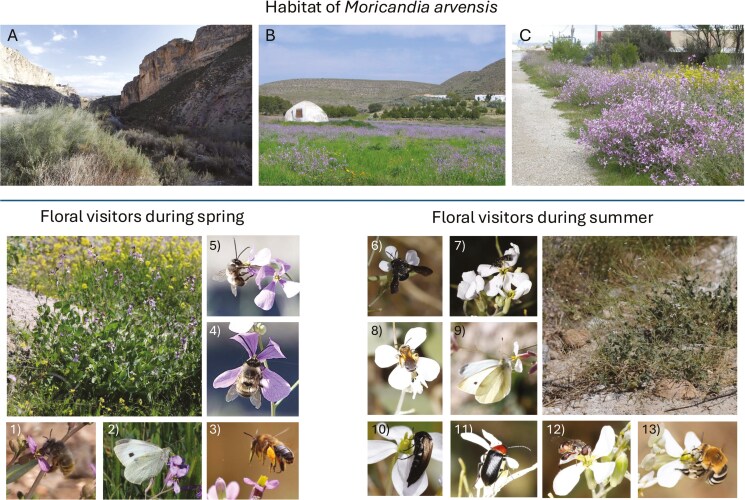
The study system. Upper panel: type of habitats occupied by *Moricandia arvensis* in SE Spain, A) Natural population (Negratín population, Granada province); B) Agricultural habitat (Agua Amarga, Almería province) and C) Industrial area (Baza, Granada Province). Lower panel: representative pollinator species of *Moricandia arvensis* in spring and summer. 1) *Anthophora plumipes*; 2) *Pieris rapae*; 3) *Anthophora* sp.; 4) *Eucera nigrilabris*; 5) *Eucera* sp.; 6) *Andrena agilissima*; 7) *Lasioglossum* sp.; 8) *Halictus* sp.; 9) *Pieris rapae*; 10) *Mordellistena* sp.; 11) *Heliotaurus ruficollis*; 12) *Syritta* sp. and 13) *Amegilla quadrifasciata*.

*Moricandia arvensis* is widely distributed in arid zones of the Iberian Peninsula, living in a wide range of habitats, from natural areas to highly anthropically disturbed areas (Fig. 1). Many phenotypic traits contribute to the seasonal floral polyphenism of *M. arvensis* (Gómez *et al*. 2020, 2022 a, 2024). Spring floral morph of *M. arvensis* is large and cross-shaped with non-overlapped petals. In contrast, the summer floral morph is smaller, with rounded corolla and overlapped petals. In addition, the spring morph is purple to the human eye, whereas the summer morph is white, with the spring morph being apparently visible to bees and the summer morph to flies (Gómez *et al.* 2020, 2024). The clear between-morph difference in colour is primarily due to the differential seasonal production of anthocyanins and flavonols (Table 1). Summer floral morph has lower herkogamy values than the spring floral morph, both when calculated with the lower whorl of stamens and when calculated with the upper whorl of stamens (Table 1). Furthermore, in both floral morphs, anthers and stigmas are exerted outside the corolla tube (Table 1). Overall, the summer morph seems to be more integrated phenotypically than the spring morph. Floral morphs also differed in the total production of flowers, with individual plants producing over 15 times more spring flowers than summer flowers (Table 1). The two morphs also differ in the amount of reward they offer. The spring morph appears to produce three times more nectar and sugar than the summer morph despite the sugar concentration being equivalent between the two morphs (Table 1). Both morphs require pollinators to have a full seed set since they did not produce seeds after autogamous hand-pollination (Table 1).

**Table 1.** Pollination traits of the two morphs of *Moricandia arvensis* (values are presented as mean ± sd). Sources: 1: Gómez *et al.* 2020; 2: Gómez *et al.* 2024; 3: Gómez *et al.* 2022a and 4: this study (**see Supporting Information Information—Method S1** for a description of the methodology used).

Pollination traits	Spring floral morph	Summer floral morph	Source
Floral traits			
Corolla tube length (mm)	12.4 ± 0.6	10.7 ± 1.4	1,4
Corolla diameter (mm)	23.0 ± 2.1	14.1 ± 2.0	1,4
Petal shape	Narrow	Wide	1
Corolla shape	Cross-shape	Rounded	1
Corolla colour (human eye)	Purple	White	1
Corolla hue^1^	245.21 ± 54.8	90.26 ± 2.59	2
Corolla brightness^2^	36.55 ± 7.40	38.94 ± 5.36	2
Corolla chroma^3^	0.05 ± 0.03	0.29 ± 0.02	2
Corolla achromatic contrast (bees)^4^	0.19 ± 0.06	0.18 ± 0.03	2
Corolla chromatic contrast (bees)^5^	0.22 ± 0.04	0.28 ± 0.04	2
Corolla chromatic contrast (flies)^6^	0.62 ± 0.04	0.28 ± 0.05	2
Petal anthocyanins (cyanidin mg g^−1^)	4.80 ± 1.06	0.30 ± 0.34	1
Petal flavonols (kaempferol mg g^−1^)	21.40 ± 5.12	82.60 ± 24.33	1
Stamens exertion	1.86 ± 0.89	1.35 ± 0.50	4
Stigma exertion	0.83 ± 0.86	0.77 ± 0.76	4
Herkogamy lower whorl of stamens (mm)	0.79 ± 0.67	0.48 ± 0.63	4
Herkogamy upper whorl of stamens (mm)	−1.02 ± 0.86	−0.58 ± 0.68	4
Floral integration (%)^7^	8.5 ± 0.9	14.6 ±3.3	1
Plant traits			
Plant height (cm)	53.70 ± 14.70	57.20 ± 19.70	2
Total number of flowers by plant	386.9 ± 671.9	24.50 ± 46.50	2
Reward traits			
Volume of nectar (µL)	0.22 ± 0.10	0.08 ± 0.03	4
Concentration of nectar (% of sugar)	20.30 ± 1.50	22.00 ± 1.50	4
Sugar quantity (mg)	0.05 ± 0.02	0.02 ± 0.01	4
Reproductive traits			
Autogamous female fertility (%)^8^	2.15 ± 13.25	0.40 ± 2.01	1
Allogamous female fertility (%)^8^	46.25 ± 38.71	23.08 ± 31.83	1
Floral evolution traits			
Divergence from *Moricandia* ancestor^9^	0.081	0.195	3
Divergence from *M. arvensis* ancestor^9^	0.024	0.287	3

^1^The dominant wavelength.

^2^The sum of the reflectance values over the entire reflectance spectrum.

^3^The difference between the maximum and the minimum values of reflectance between the average reflectance of the spectrum.

^4^The degree to which each petal colour loci generates an excitation value different from 0.5 in the green receptor using the colour hexagon model for bees.

^5^The distance of each petal colour loci to background in the colour hexagon model for bees.

^6^The distance of each petal colour loci to background in the colour hexagon model for hoverflies.

^7^The variance of eigenvalues of the covariance matrix of the floral traits.

^8^Autogamy and allogamy was quantified by hand-pollinating plants in a controlled (greenhouse) environment.

^9^The Euclidean distance from each ancestor in a Brassicaceae phylospace.

### Pollinator assemblage of spring and summer floral morphs

We have recorded the insects visiting the flowers of *Moricandia arvensis* during 8 years (2016–2023) in six localities of SE Spain: Baza (Granada province, 37° 30.5ʹN; 2° 40.9ʹW), Quesada (Jaén province, Spain, 37° 48.3ʹN; 3° 03.4ʹW), Malaha (Granada province, 37°08.4ʹN; 3°43.9ʹW), Negratín (Granada province, 37° 33.7ʹN; 3° 57.5ʹW), Olula del Rio (Almería province, 37° 23.3’N; 2° 17.9ʹW) and Tabernas (Almería province 37° 00.3ʹN, 2° 27.4ʹW). Censuses of floral visitors were carried out in each locality between 11:00 am and 5:00 pm both during the blooming period when spring floral morphs are displayed (February–March) and during the blooming period when the summer floral morphs are displayed (June–August). In each survey, we recorded for 2 h those insects contacting anthers or stigma and doing legitimate visits at least during part of their foraging in flowers. We only counted those insects contacting anthers or stigma, meaning that several species of petal eaters, predispersal seed predators and nectar thieves/robbers were not included in the surveys. Previous studies using the same methodology carried out with similar Brassicaceae species and performing rarefaction analysis indicate that a sample of 130–150 insects provides an accurate estimate of the diversity of pollinator assemblages (Gómez *et al*. 2007). Each survey was conducted at least by two researchers simultaneously, sampling each population for at least 10 h/person. Unfortunately, we could not survey all populations during all years.

Because *M. arvensis* is a generalist species (Gómez *et al*. 2020, 2024), the relevant interaction units are functional groups, defined as groups of pollinators that behave in similar ways on a flower and exert similar selection pressures (Fenster *et al*. 2004). For this reason, we grouped all pollinators visiting *M. arvensis* into functional groups employing the same classification utilised in previous studies (Gómez *et al*. 2020, 2022*a*) that is based on similarity in traits related to the selection pressures they exert, such as body length, proboscis length, morphological match with the flower, foraging behaviour and feeding habits (Gómez and Zamora 1999; Fenster *et al*. 2004) (see **Supporting Information—**Table S1 for the description of the functional groups visiting *M. arvensis*).

The generalisation of the pollination system of each floral morph was estimated by means of the alfa and beta diversities of their pollinator assemblages. Alfa diversity was calculated using the Probability of Interspecific Encounter (PIE) of Hurlbert’s (1971) as


$$\begin{array}{l} PIE=\left(\frac{N}{N-1}\right)\left(1-\;\underset{i=1}{\overset{P}{\sum }}\;p_{i}^{2}\right) \end{array}$$

where *N* is the total number of individuals and *p*_*i*_ is the relative abundance of functional groups *i*. It indicates the probability that two insects visiting the same floral morph belong to different functional groups. It is an evenness index that combines two factors that affect diversity, dominance and abundance. We compared the magnitude of the alfa diversity of the pollinator assemblage between floral morphs by generating bootstrapped estimates of the indices, obtaining their 95 % confidence intervals and checking their overlap (Magurran 2004; Chao *et al*. 2014). This analysis was performed using the function ChaoSimpson from the R library ‘iNEXT’ (Hsieh *et al*. 2024). Beta diversity was estimated as the variation in the composition of functional groups between floral morphs (Baselga and Orme 2012). For this, we tested if there were differences in composition between seasons using a Permutational Analysis of Variance (Anderson 2001). This analysis was performed using the function adonis2 in the R library ‘vegan’ (Oksanen *et al*. 2022), including population as a covariable to control for between-population variation in pollinator fauna. In addition, we performed a Principal Coordinate Analysis (PCoA) to test whether the floral morph expressed during each season by each population plays a role in the clustering of populations according to their pollinators, and to explore which functional group of pollinators was most correlated with this clustering. (Borcard *et al*. 2011). The PCoA was performed using the function cdmscale in the R library ‘vegan’ using Bray–Curtis dissimilarities as input matrix (Oksanen *et al*. 2022). The projection of each pollination functional group onto the resulting two-dimensional space was performed using the function add.spec.scores in the R library ‘BiodiversityR’ (Kindt 2005).

### Pollinator preference experiments

We experimentally determined the preference of pollinators for the two plastic morphs of *M. arvensis* by simultaneously offering them in spring and summer. To do so, we performed an experiment in the field in which, for 5 min, we simultaneously offered 10 plants bearing spring floral morphs and 10 plants bearing summer floral morphs randomly distributed in 5 × 4 grids. Experimental plants were separated 1 m from each other to avoid overlap of inflorescences. During each trial, we recorded the number and identity of pollinators visiting the flowers of each experimental plant. We randomly redistributed the position of each plant in the grid before starting a new trial and changed the plants after 10 trials. Experimental plants were similar in size and floral display. However, to avoid any confounding effect, we recorded the total number of open flowers displayed by each experimental plant during each trial. Experimental plants were grown in the climate-controlled greenhouse under each environmental condition (spring and summer) and taken daily to the field in pots. Each trial was done by two researchers simultaneously and was performed between 12:00 and 14:00 local time in spring and between 11:00 and 13:00 local time in summer. The experiments were performed over 2 years (2019 and 2020) in five natural populations of *M. arvensis* (Baza, Quesada, Malaha, Negratín, Tabernas). In total, we carried out 150 trials in 2019 and 216 trials in 2020, totalling 12.5 h and 18 h, respectively. We first compared the pollinator assemblage of the experimental plants displaying different floral morphs by means of a Permutational Analysis of Variance (Anderson 2001), by using the function adonis2 in the R library ‘vegan’ (Oksanen *et al*. 2022).

The preference of each pollinator functional group visiting the flowers of the experimental plants was estimated using the Jacobs’ D index (Jacobs 1974), a modification of the Ivlev’s E electivity index (Ivlev 1961) that is robust to dramatic changes in the relative abundance of the resources (Lechowicz 1982)


$$\begin{array}{l} D_{i}=\frac{r_{i}-p_{i}}{r_{i}+p_{i}-2r_{i}p_{i}} \end{array}$$

where *r*_*i*_ is the proportion of the food *i* in the diet and *p*_*i*_ is the proportion of diet *i* in the environment. Applied to our experiment, this index compares the relative availability of flowers of each floral morph in the environment and their relative visits by each functional group. The *D* index takes a value of zero under random feeding and deviates symmetrically from zero between plus and minus one for preferred and avoided items, respectively. In an environment with only two food types, like the one used here (two floral morphs), Jacob’s index can potentially give the full range of values (−1 ≤ *D* ≤ 1) for any particular value of floral visitation and availability (Lechowicz 1982). Jacob’s *D* index was calculated using the function ivlev in the R library ‘selectapref’ (Richardson 2020). The statistical significance of the preferences was found by testing for significant deviation from random visitation rate using a test for goodness of fit (Lechowicz 1982). If pollinator functional groups visit flowers at random, the null hypothesis is that the number of visits to a given flower morph by a given pollinator functional group should be directly proportional to the relative abundance of that morph (Lechowicz 1982).

### Pollination effectiveness

The quantity component (QTC) of the pollination effectiveness was expressed in this study as the number of visits made by each pollination functional group to the flowers of *M. arvensis* per plant and hour. QTC was assessed in the Negratín population during 2019. For this, we marked 100 co-occurring plants in February before flowering. When the plants started flowering, we conducted floral visitor surveys, in which we recorded insects visiting the flowers of each individual plant for 5 min. We only recorded insects that came into contact with the anthers or stigma and made legitimate visits, at least during part of their foraging on the flowers. Insects that ate petals or stole nectar without making any legitimate visits were not recorded as pollinators. All surveys were carried out by the same researcher between 11.00 and 17.00 h. Surveys were conducted both during the period when plants were displaying spring flowers (early March to early May) and during the period when they were displaying summer flowers (late May to early July), on a regular basis (approximately every 2 weeks).

The quality component (QLC) of the pollination effectiveness was expressed in this study as the number of seeds produced per visit by each pollination functional group. This means that we are only calculating the contribution of pollinator functional groups to the female fitness of *Moricandia arvensis*. To assess the QLC component of different functional groups on spring and summer flowers, unvisited virgin spring and summer flowers were exposed to floral visitors from different functional groups. The plants used in this experiment were maintained in the greenhouse under spring or summer conditions until they were presented to floral visitors in experimental arenas located in two natural populations (Malaha and Negratín populations). This prevented the flowers from being visited before their exposure in the arena. Immediately after each flower was visited, its petals were removed to prevent revisitation, and it was individually marked to identify the functional group of the visitor. At the end of the exposure period, the plants were returned to the greenhouse, and fruit and seed production was awaited. In the laboratory, the number of flowers that set fruits, the number of viable seeds per fruit, the number of unfertilised ovules and the number of aborted seeds were counted under a magnifying glass. This allowed the calculation of the qualitative component of the effectiveness of each functional group on each floral morph.

To explore whether pollinators can be grouped according to their effectiveness, we checked the spatial distribution of the effectiveness values across the effectiveness landscape generated by the two components (Gómez *et al*. 2022*b*). A clumped spatial pattern indicates that there are distinct groups of species that have similar effects on plant fitness, revealing the occurrence of functional equivalence within clustered groups of interacting organisms (Calviño-Cancela and Martín-Herrero 2009; González-Castro *et al*. 2015; Palacio 2019). In contrast, over-dispersed effectiveness landscapes indicate that different pollinators have very distinct effects on plant fitness, and consequently, they are not interchangeable. The spatial pattern of effectiveness values was tested with the Clark–Evans R test (Wiegand and Moloney 2014) with Donnelly edge correction (Baddeley *et al*. 2015) and the Hopkins–Skellam A index (Baddeley *et al*. 2015) to control for spatial inhomogeneity. If the landscape is clustered, the number of clusters was found using a hierarchical cluster analysis by means of the function NBClust in the R package NbClust that determines the optimal number of clusters by choosing the most frequent partition obtained from 30 validation indices (Charrad *et al*. 2014). We also checked for the correlation between QLC and QTC, assuming that a positive correlation is associated with specialised interactions (Gómez *et al.* 2022 b).

## Results

### Differences in pollinator assemblage between spring and summer floral morphs

A total of 6729 insects belonging to 29 pollinator functional groups were detected visiting the flowers of *Moricandia arvensis* in the six studied populations during the study period (Table 2). Flowers were visited in spring by 23 functional groups, whereas in summer they were visited by 25 functional groups (Table 2). The diversity of functional groups visiting the flowers was much lower in spring (0.588 ± 0.009, Boostrapped CI = 0.588–0.606; Chao-estimated Hurlbert PIE) than in summer (0.802 ± 0.008, Boostrapped Confidence Interval = 0.802–0.819).

**Table 2.** Abundance of pollinators (in percentage) visiting the flowers of *Moricandia arvensis* in six populations of South East Spain during spring and summer. Negr = Negratin, Mal = Malaha, Baza = Baza, Ques = Quesada, Tab = Tabernas and Olu = Olula

		Spring							Summer						
Code	Functional Group	Negr	Mal	Baza	Ques	Tab	Olu	Total	Negr	Mal	Baza	Ques	Tab	Olu	Total
	Hymenoptera														
1	Long-tongued large bees	64.6	40.7	70.5	97.9	66.7	64.6	61.8	16.2	1.7				1.0	15.5
2	Long-tongued medium-sized bees		16.7					2.4							
3	Short-tongued large bees	2.1	1.8	9.1		1.3	3.6	3.0	13.7	1.0				0.6	6.6
4	Short-tongued medium-sized bees	1.8	2.4	0.9			1.4	1.6	3.6						1.7
5	Short-tongued small bees	8.3	1.0	8.5		9.6	4.1	6.0	36.0	44.1		57.1		42.4	36.2
6	Short-tongued extra small bees	0.4						0.2	6.8						3.1
7	Honeybees		2.9				1.5	0.9							
8	Pollen wasps	0.1						0.0							
9	Large nectar-feeding wasps								0.2						0.1
10	Small nectar-feeding wasps	1.4						0.6	0.3						0.1
11	Large ants	0.9						0.4	6.4						2.9
12	Small ants								0.2						0.1
	Diptera														
13	Hovering beeflies	6.6	11.7			20.8	9.8	8.5		7.6		28.5	16.7	14.8	6.8
14	Non-hovering beeflies		0.4				0.2	0.1	0.4						0.2
15	Long-tongued flies								0.2						0.1
16	Large hoverflies		0.4				0.2	0.2	0.1	2.4				1.4	0.7
17	Small hoverflies	0.1	1.9	3.4	2.1	0.8	2.3	1.3	3.3	12.8				7.6	5.0
18	Large flies		2.8			0.8	1.6	0.9	0.5						0.2
19	Small flies		0.7				0.4	0.2	0.2						0.1
	Lepidoptera														
20	Large butterflies	1.5	6.3	2.5			3.9	2.9	2.8	22.9				13.5	7.6
21	Small butterflies		0.6				0.3	0.2	0.2						0.1
22	Hawkmoths	0.8	4.9				2.5	1.8							
23	Small moths		0.1				0.1			2.4				1.4	0.7
	Coleoptera														
24	Large beetles	3.2	1.8	0.3			1.0	2.0	1.8				66.7	8.2	4.6
25	Small beetles	0.7	0.9				0.	0.6	3.9	2.8				1.6	2.6
26	Small diving beetles	7.2	1.9				1.0	3.7	1.1	0.7		7.1		2.5	1.7
	Others														
27	Aphids								0.1						0.0
28	Bugs								1.4						0.7
29	Thrips			4.7			1.1	0.6	0.4	1.4		7.1	16.7	4.9	2.5
	Total	2036	678	319	194	240	1318	4625	968	288	0	140	60	488	2104

**Figure 2.**
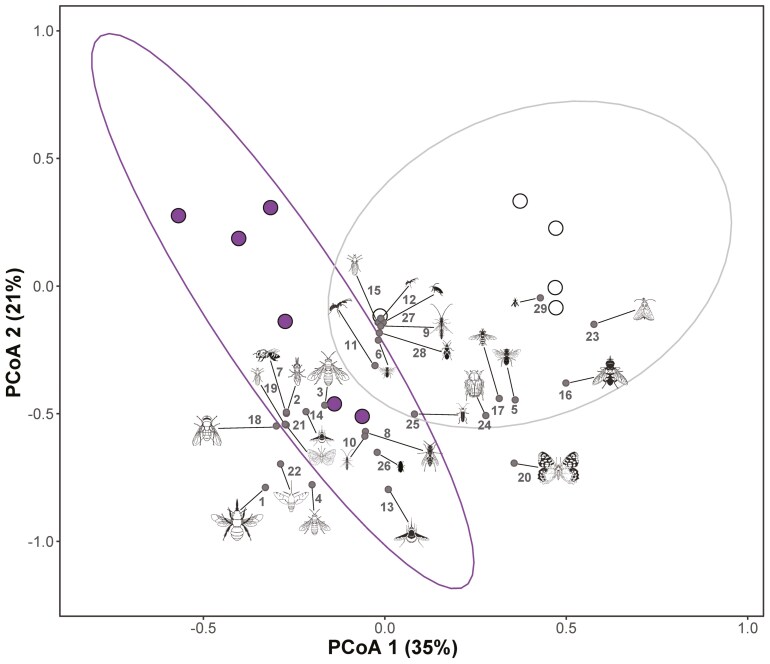
Principal coordinates analysis. Results of PCoA showing the first two principal coordinates that, together, explain 56% of the variation in pollinator assemblage. Each dot is a site × season combination (spring surveys in purple, summer surveys in white) whereas the projection of each pollinator functional group is shown in grey (numbers correspond to the code of each pollinator functional group as appears in Table 2). Insect silhouettes were drawn by Divulgare (divulgare.net) under a Creative Commons license (http://creativecommons.org/licenses/by-nc-sa/3.0).

The composition of the visitor assemblage did not differ among populations (*F*= 0.91, *P* = 0.604, Permutational Analysis of Variance). However, it differed between seasons (*F*= 3.66, *P* = 0.004, Permutational Analysis of Variance). This outcome agrees with the PCoA, which shows that season was the main factor in grouping the populations in the two-dimensional space (Fig. 2). This ordination analysis showed that the pollinator assemblages differed between spring and summer. During spring, the most frequent floral visitors were long-tongued large bees belonging to the Anthophorini tribe, mostly several species of *Anthophora* (*Anthophora plumipes*, *A. leucophaea*, *A. dispar*, etc.), *Amegilla* (such as *Amegilla quadrifasciata*) and *Eucera* (such as *Eucera longicornis*) and, to a lesser extent, long-tongued hovering beeflies belonging to the genus *Bombylius,* and short-tongued large bees belonging to the genera *Halictus* and *Andrena* (Figs. 1 and 2). During summer, the composition of the pollinator assemblage changed, and the most frequent visitors were short-tongued small bees, long-tongued large bees and large butterflies mainly belonging to the family Pieridae (such as *Pieris rapae*, *Pieris brassicae*, *Euchloe crameri*, *Colias alfacariensis* or *C. croceus*) (Table 2; Figs. 1 and 2). During this hot season, the flowers of *M. arvensis* were also visited by many small insects such as large beetles (mostly Cetoniidae, Alleculinae, Scarabeidae Cleridae and Meloidae) and small beetles (like Melyridae, Bruchidae, Mordellidae or Dermestidae), hoverflies (mostly *Eupeodes corollae*, *Sphaerophoria* spp. and *Eristalis* spp.), ants and thrips (Table 2; Figs. 1 and 2). In general, the assemblage of floral visitors was dominated by nectar-feeding long-tongued insects during spring but by pollen-eating short-tongued insects during summer.

### Pollinator preference experiments

The flowers of the experimental plants were visited by 16 functional groups of pollinators, which in total made 2884 floral visits **[see Supporting Information—Table S2]**. The pollinator assemblage differed among seasons (*F*= 3.36, *P* = 0.036, Permutational Analysis of Variance). However, neither the floral morphs (*F* = 1.15, *P* = 0.358) nor the interaction term (*F*= 0.07, *P* = 0.938) affected the composition of the pollinator assemblage visiting the experimental plants. In general, the seasonal change in the pollinator assemblage of the experimental plants was similar to that observed in the plants belonging to the natural populations. So, whereas long-tongued large bees were the most abundant floral visitors during spring, short-tongued small and large bees as well as butterflies and beeflies were particularly abundant during summer **[see Supporting Information—Table S3**].

**Figure 3.**
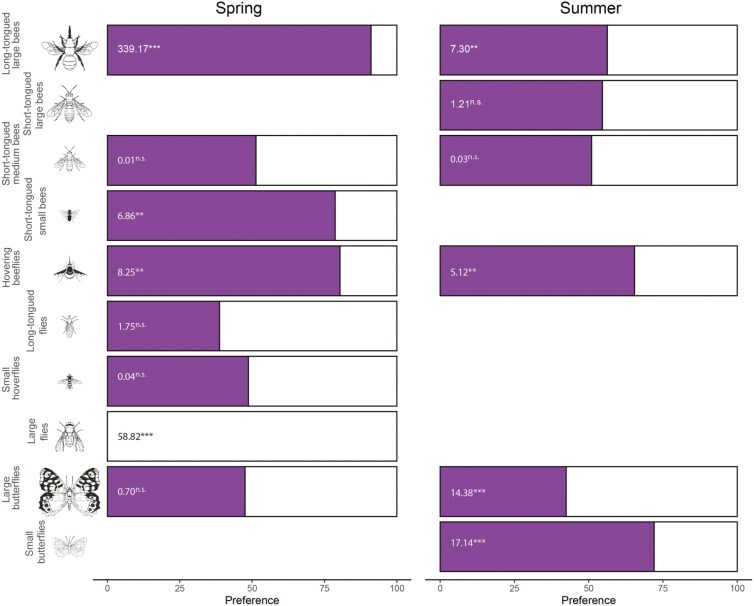
Preference of pollinators. Percentage of visits made by each pollinator functional group to the spring (purple colour) and summer (white colour) floral morphs offered in the preference experiments. Figures are the results of the goodness of test testing departure from random preference (n.s. = non-significant, **P < 0.001, ***P < 0.0001). We show here only those pollinator groups with enough sample size (**see Supporting Information—Methods** for details and **Table S3** for the results of all functional groups tested). Insect silhouettes were drawn by Divulgare (divulgare.net) under a Creative Commons license (http://creativecommons.org/licenses/by-nc-sa/3.0).

Pollinator functional groups differed in their preferences for floral morphs. Using only the 10 functional groups with large enough sample sizes (**see Supporting Information—Table S3** to see the results of the 16 functional groups included in the experiment), it can be observed that some pollinator groups, such as long-tongued large bees, short-tongued small bees, hovering beeflies or small butterflies, prefer to visit spring floral morphs either in spring or in summer (Fig. 3). In contrast, other pollinator groups, such as large flies and large butterflies, visited the summer floral morphs more frequently than expected by chance (Fig. 3).

### Pollination effectiveness

**Figure 4.**
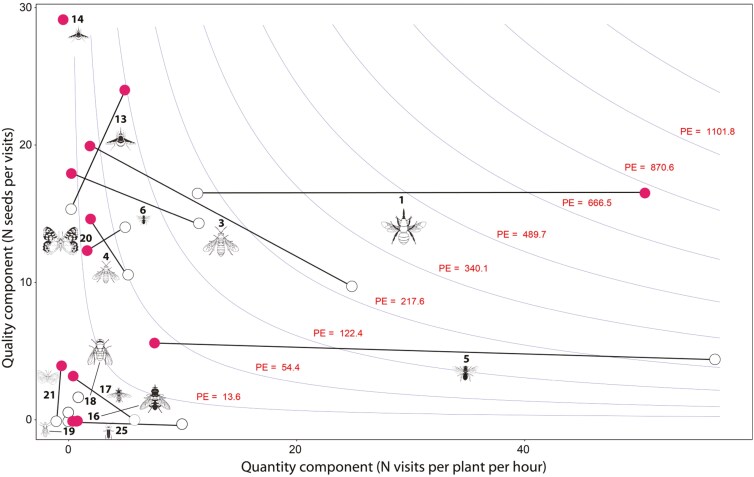
Pollination effectiveness. Effectiveness landscape of main functional groups visualised as a combination of quality (number of seeds produced per visit) and quantity (number of visits made to flowers in a population during the blooming period) components. It is shown the effectiveness of each pollinator functional group when visiting spring (purple dots) and summer (white dots) floral morphs. The numbers correspond to the code of each pollinator functional group as appears in Table 2. Insect silhouettes were drawn by Divulgare (divulgare.net) under a Creative Commons license (http://creativecommons.org/licenses/by-nc-sa/3.0).

A total of 852 experimental virgin flowers were visited by insects belonging to 13 pollinator functional groups **[see Supporting Information—Table S2]**. Pollinator functional groups greatly differed in their effectiveness as pollinators, estimated as the number of seeds produced per plant and hour, both when visiting spring flower morph (Deviance = 848.3, *P* < 0.0001, binomial GLM) and when visiting summer flower morph (Deviance = 390.2, *P* < 0.0001, binomial GLM) (Fig. 4; **see Supporting Information—Table S4**). The most effective pollinators of spring floral morph were long-tongued large bees and, to a lesser extent, hovering beeflies (Fig. 4), their activity at flower mediating the production of about 78 % and 11 % of seeds produced by this morph, respectively, the rest of the functional groups contributing less than 5 % **[see Supporting Information—Table S4]**. In contrast, the effectiveness values of functional groups were more evenly distributed in summer flowers (Fig. 4), with 26 % of the seed production mediated by the activity of short-tongued small bees, 19 % by long-tongued large bees, 17 % by short-tongued extra small bees and 19% by long-tongued large bees **[see Supporting Information—Table S4]**. Due to the difference in the proportional contribution of each functional group, floral morphs also differed in the proportion of ovules passing to seed when visited by a pollinator (Deviance = 106.2, Estimate = −0.25, *P* < 0.001, binomial General Linear Model (GLM)), with spring morphs maturing to seed 24.6 ± 1.5 % (mean ± 1 standard error (se) ) of the ovules and summer morphs maturing 20.7 ± 1.8 %.

The effectiveness of most pollinator functional groups varied between floral morphs (Deviance = 106.2, Estimate = −0.25, *P* < 0.001, binomial GLM) (Fig. 4, **see Supporting Information—Table S4**). In some functional groups, such as hovering beeflies or small butterflies, the change in effectiveness between floral morphs was due to a change in their values in QLC (Fig. 4). In other functional groups, such as short-tongued large bees or extra small bees, this change was caused by a combination in the value of both components QLC and QTC (Fig. 4). However, the between-morph change in effectiveness of the two most effective functional groups, long-tongued large bees in spring floral morphs and short-tongued small bees in summer floral morphs, was due to a strong change in their value of the QTC (Fig. 4). That is, these two functional groups produced a similar number of seeds when visiting a single flower of either floral morph, but differed significantly in their abundance in the flowers of each morph.

The effectiveness landscape (Fig. 4) was significantly clumped both in spring (Clark–Evans *R* index = 0.47, *A* = 0.20, *P* < 0.01, 1000 bootstrapping iterations) and in summer (Clark–Evans *R* index = 0.52, *A* = 0.77, *P* < 0.05). In all cases, and according to the majority rule, the number of clusters in the landscape was 3. One cluster was composed of pollinators with very low effectiveness in both floral morphs (Fig. 4; **see Supporting Information—Fig. S1**). Another cluster was composed of pollinators with intermediate effectiveness, beeflies for spring floral morph and large butterflies for summer floral morph (Fig. 4, **Supporting Information—Fig. S1**). The third cluster was composed of pollinators with high effectiveness, long-tongued large bees for spring floral morph and short-tongued bees for summer floral morph (Fig. 4, **see Supporting Information—Fig. S1**).

There was no significant correlation between the QTC and QLC components (spring: *r* = 0.18, *P* = 0.559; summer: *r* = 0.05, *P* = 0.887; Pearson correlation).

### Diversity of the pollination system when considering effectiveness

**Figure 5.**
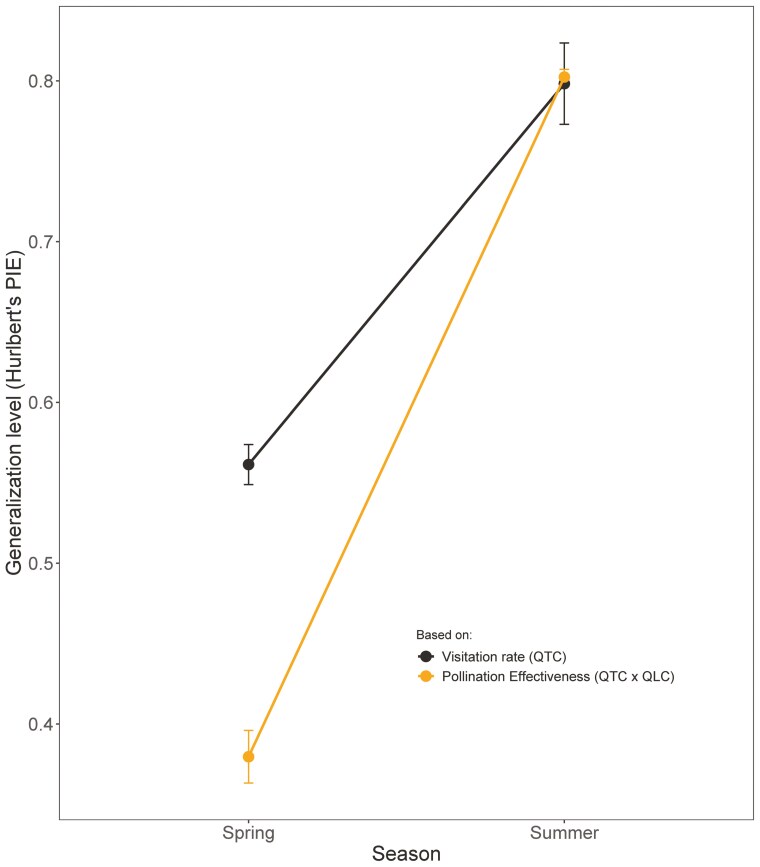
Generalisation degree of pollination systems. Values of Chao-estimated Hurlbert’s PIE diversity index (mean ± 1 s.e.) estimated using the visitation rate of pollinator functional groups (in black) and the number of seeds produced by each functional group (in orange).

The diversity of the pollination system of *Moricandia arvensis* was significantly lower during spring when calculated using the effectiveness of each pollinator functional group (calculated as number of seeds produced per plant) (Chao-estimated Hurlbert PIE on effectiveness: 0.49 ± 0.002, Bootstrapped CI= 0.58–0.54) than when calculated using only their visitation rate (Fig. 5; Chao-estimated Hurlbert PIE on effectiveness: 0.56 ± 0.01, Bootstrapped CI= 0.490—0.495). However, during the summer the opposite was true, and diversity was slightly higher when calculated using the effectiveness (Fig. 5; Chao-estimated Hurlbert PIE: 0.80 ± 0.005, Bootstrapped CI= 0.801—0.799) than when calculated using visitation rate (Chao-estimated Hurlbert PIE: 0.79 ± 0.02, Bootstrapped CI= 0.75—0.0.845). Consequently, the between-season difference in diversity was greater when seed production was used than when only floral visits were used (Fig. 5).

## Discussion

*Moricandia arvensis* is characterised by being the only known plant species in which the same individuals can produce two radically different but equally integrated and functional floral forms in different seasons, large purple cross-shaped during spring and small white and rounded during summer (Gómez *et al*. 2020). This within-individual floral polyphenism seems to benefit *M. arvensis*, since plants displaying two floral morphs produce more seeds (Gómez *et al*. 2020). In addition, floral polyphenism is widely extended all over the entire distribution of *M. arvensis* (Perfectti *et al*. 2017; Gómez *et al*. 2024). This suggests that floral polyphenism is ancient in the *M. arvensis* lineage and has not been eliminated during its evolutionary history (Gómez *et al*. 2024).

A major consequence of this seasonal within-individual floral polyphenism is that the same individual plants could interact with two contrasting pollinator assemblages (Gómez *et al*. 2020, 2022a). Our study confirms this idea and shows that the pollinator assemblages visiting the spring flowers significantly differed in composition from those visiting the summer flowers, even within the same localities. This variation is even more remarkable if we take into account that we compared functional groups rather than species. Spring flowers were mainly visited by long-tongued bees (Apidae: Anthophorini) and other long-tongued insects. These functional groups are the main pollinators of most relatives of *M. arvensis*, such as the other *Moricandia* species (Dukas and Shmida 1989; Küchmeister *et al*. 1995; González-Megías 2016; Gómez *et al*. 1996, 2020), *Rytidocarpus moricandioides, Eruca vesicaria* or *Eruca foleyi* (Gómez *et al*. 2016, 2022 a; Barazani *et al*. 2019; Shakeel *et al*. 2019; Sentil *et al.* 2022). In contrast, summer flowers of *M. arvensis* were visited by a mixture of short-tongued insects, such as short-tongued small bees, hoverflies and beetles, and long-tongued insects, such as long-tongued bees, butterflies or beeflies. During the summer season, the pollinator assemblage of *M. arvensis* resembles more those of distant Brassicaceae species belonging to other tribes, such as *Alliaria* (Cruden *et al*. 1996), *Arabis* (Strzalkowska-Abramek *et al*. 2016), *Cardamine* (Salisbury 1965), *Erysimum* (Pesenko *et al.* 1980; Gómez *et al.* 2014, 2015), *Hormathophylla* (Gómez and Zamora 1999), *Lepidium* (Robertson and Leavitt 2011), *Lobularia* (Gómez 2000), *Parrya* (Fulkerson *et al*. 2012), *Streptanthus* (Preston 1994) or *Strigosella* (Pesenko *et al.* 1980). Significant variation in the composition of the pollinator fauna has been described in many plant species both between populations and between years (Herrera 1988; Horvitz and Schemske 1990; Guitián *et al*. 1996; Traveset and Sáez 1997; Gómez and Zamora 1999; Fenster and Dudash 2001; Price *et al*. 2005; Dupont *et al*. 2009; Castro *et al*. 2013; Gómez *et al*. 2014; Zych *et al*. 2019). Variation in pollinator assemblage composition has also been found between seasons (Cane and Payne 1993; Fisogni *et al*. 2016; Valverde *et al*. 2016) and between co-occurring individuals occupying different microsites (Herrera 1995; Prado *et al*. 2021). In our case, this variation is more extreme, since floral polyphenism allows the same individuals of *M. arvensis* to explore two contrasting pollination niches (Gómez *et al*. 2022*a*).

The observed between-season change in the pollinator faunas does not mean that *M. arvensis* is seasonally specialised. On the contrary, we found that, due to the marked seasonal differences in both the form of the flowers and the type of pollinators, the diversity of pollination systems changed dramatically between seasons. While the probability of being visited consecutively by two insects belonging to different functional groups was 59 % for a spring flower, this probability rose to 80 % for summer flowers. Because most Brassicaceae display a highly generalised floral bauplan (Nikolov 2019; Gómez *et al*. 2022 a), most species of this family tend to interact with diverse pollinator communities and have broad pollination niches (Gómez and Zamora 1999; Robertson and Klemash 2003; Gómez *et al*. 2007, 2022a; Sahli and Conner 2007; Albrecht *et al*. 2012; Roy *et al*. 2014; Shakeel *et al*. 2019; Yadav *et al*. 2022; Streher *et al*. 2024). In this sense, many Brassicaceae are generalist species (*sensu*  Waser *et al*. 1996; Olesen *et al*. 2002). *Moricandia arvensis* exceeds these standard levels of generalisation since the same individuals bear two different floral morphs, each visited by different insects belonging to different pollination niches. So, *M. arvensis* is generalist both in spring and in summer, and emerges as a doubly generalist species. That is, the extremely generalisation level of the pollination systems of *M. arvensis* arises from the synergistic effect of high alpha (within-season) and beta (between-season) diversity.

The seasonal difference in the pollination niche could result from pollinators showing differential preferences for each flower type or as a by-product of the different availability of pollinators in each season. The evolutionary consequences of each process are radically different. Environmentally mediated heterogeneity in floral visitors causes unpredictable fluctuations in the selection undergone by plants (Siepielski *et al*. 2009; Bell 2010), a process that reduces the effectiveness of pollinator-mediated selection and limits floral evolution in response to interactions with pollinators (Schemske and Horvitz 1989; Szenteczki *et al*. 2021), favouring the existence of non-adaptive intraspecific floral trait variation (Schemske and Horvitz 1989; Fishbein and Venable 1996; Bell 2010). In contrast, when pollinators that vary temporally in abundance respond differently to floral traits, they can lead to the adaptive evolution of temporally variable phenotypes. In this scenario, plants might display each season the floral phenotype preferred by the available pollinators. However, we have found that the floral morph most preferred by pollinators was the spring morph, the summer morph being always avoided when offered simultaneously with the spring morph except for large flies and large butterflies. Most importantly, pollinators did not change their preference patterns between seasons. These findings suggest that the *M. arvensis* floral polyphenism is not shaped by pollinator-mediated selection but is only the direct consequence of environmental heterogeneity. This result is consistent with previous studies showing that floral polyphenism is counter-selected in this species due to pollinator action (Gómez *et al*. 2024). Therefore, the seasonal variation in the *M. arvensis* pollinator assemblages is decoupled from the plastic variation of its flowers and is probably merely a consequence of a change in environmental conditions filtering the pollinator community. The high generalisation level observed in the pollination system of *M. arvensis* occurs because individuals of this species flower in two contrasting fine-grained environments during their lifetime, not because plants have evolved disparate types of flowers to interact with different pollinators each season.

### The effect of floral phenotypic plasticity on pollination effectiveness

Our effectiveness analysis shows that the effectiveness landscape is clumped because the insects visiting the flowers of *M. arvensis* are grouped according to their combination of per-visit efficiency and abundance at flowers. Clumped spatial pattern of effectiveness landscapes indicates that distinct groups of species have similar effects on the fitness of their partner, revealing the occurrence of functional equivalence within clustered groups of interacting organisms (Gómez *et al*. 2022*b*). Whereas in dispersed landscapes, there is a possibility to respond to the selection exerted by those individual species having the highest fitness effects, in clumped landscapes, natural selection will favour the use of those groups of functionally equivalent species that have, altogether as a group, the highest effect on fitness, favouring the evolution of generalisation (Zamora 2000; Gómez and Zamora 2006). In *M. arvensis*, a clumped effectiveness landscape occurred both for spring and summer floral morphs, explaining at least partially why the pollination system remains generalist regardless of the floral morph displayed by the plants. In both seasons, there was a group of pollinators with a negligible effect on fitness sharing the flowers with another group with a high effect on fitness, the use of which were surely favoured by selection.

The pollinator species in the low-effectiveness cluster were the same in both floral morphs, mostly flies and hoverflies, beetles and other similar short-tongued small insects. The low effectiveness obtained by these insects resulted from the combination of their low abundance at flowers and their low per-visit efficiency. Beetles seem to be ineffective pollinators of similar Brassicaceae species (Gómez and Zamora 1999; Phillips *et al*. 2018). However, the role of flies and hoverflies as pollinators of other similar Brassicaceae remains uncertain because some studies have found that they are ineffective pollinators (Rader *et al*. 2013), whereas other studies have found that they can have moderate to high per-visit efficiency (Jauker and Wolter 2008; Ali *et al*. 2011; Jauker *et al.* 2012; Phillips *et al*. 2018). The very low per-visit efficiency that we found in our study could be caused by their inability to remove and/or deposit pollen during the floral visits. In fact, it seems that flies deposit less pollen per visit than bees in a wide range of plant species (Földesi *et al*. 2021). However, it can also be explained, at least partially, by the fact that we calculated this component of the pollinator effectiveness not as the number of grains deposited (the most frequent variable used in previous studies; but see Jauker *et al*. 2012) but as the number of seeds produced, a component of the plant reproductive success strongly influenced by the allogamous *versus* geitonogamous/autogamous origin of the pollen grains (Matsuki *et al*. 2008), something that can be especially important in our self-incompatible species. Thus, we assume that flies and beetles were probably irrelevant to seed production of *M. arvensis* in either season, not only because they deposit little pollen but also because they move more frequently between flowers of the same plant and produce geitonogamous pollination more often than bees, beeflies and butterflies.

In contrast, the identity of the effective pollinators varied between seasons. And this was apparent even though we worked with functional groups rather than with individual species. The most effective pollinators of spring flowers were long-tongued large bees, which produced about two thirds of the seeds in the plant populations, followed by the beeflies, which produced about 15 % of seeds. That is, during spring, over 90 % of the seeds of any SE Spain plant population were produced by insects with long proboscides. The predominance of these two groups decreased considerably during the summer, a season in which the importance as pollinators was evenly distributed between insects with long proboscides, like the two of them, and butterflies and insects with short proboscides, like several species of large and small solitary bees. It is noteworthy that the functional group with the highest effectiveness each season (long-tongued large bees in spring and short-tongued small bees in summer) had similar per-visit efficiency in each floral morph; their change in effectiveness being exclusively caused by a strong seasonal variation in visitation rates. In contrast, the between-morph change in the effectiveness value of the rest of the effective pollinators (hovering beeflies, large butterflies and short-tongued large bees) was produced by a change in per-visit efficiencies rather than by a change in their abundance at flowers. Interestingly, in practically all cases, these changes occur in the sense of loss of efficiency in seed production of the summer morph. This suggests that the floral polyphenism of *M. arvensis* probably entails changes in some floral traits that disrupt the match between the flower and those pollinators by influencing the behaviour and mechanical fitting of some pollinators at flowers (Neal *et al*. 1998). For example, because *M. arvensis* seems to be highly self-incompatible (Table 1), those factors favouring self-pollen transfer could contribute to decrease pollination efficiency in summer floral morphs (Valverde *et al.* 2019). In this regard, the sexual structures of summer flowers are less exerted than those of spring flowers and have a lower degree of herkogamy, two characteristics that increase the probability of pollinator-facilitated self-pollination (Dai and Galloway 2011) and magnify the cost of hermaphroditism in self-incompatible species (Barrett 2002; Waites and Agren 2006). This might explain why the per-visit efficiency of most insects is higher when visiting spring than when visiting summer flowers. Likewise, because visitation duration has been shown to increase pollination efficiency in some plant species (Fishbein and Venable 1996; Ivey *et al*. 2003), those traits increasing the length of the visits of the pollinator may act to augment their per-visit efficiency. Several studies have shown that increasing the amount of nectar increases the frequency of insect visits and lengthens the duration of each visit, concomitantly increasing the efficiency of the visits (Pleasant 1983; Real and Ratchke 1988; Manetas and Petropoulou 2000; Li *et al*. 2021). Spring flowers of *M. arvensis* produced about three times as much nectar and sugar quantity as summer flowers (Table 1), which may also help explain the between-season difference in per-visit efficiency of some insects. In addition, flower shape also influences the per-visit efficiency of some pollinators (Conner *et al*. 1995; Wu and Li 2017). For example, hovering beeflies spend more time visiting the flowers of the crucifer *Erysimum mediohispanicum* that have large petals and provide a good landing platform because they tend to collect nectar while standing on their second and third pair of legs (Gómez *et al*. 2008). The observed increase in the per-visit efficiency of hovering beeflies on spring flowers could be due, at least in part, to the fact that this morph has longer petals and may provide a better landing platform than summer flowers.

### Taxonomic versus functional generalisation of pollination systems

Most studies exploring the generalisation–specialisation level of pollination systems do not include information on the role as pollinators of those animals visiting the flowers. In these circumstances, concluding whether a given plant species is a generalist based on the number of visitors alone could be misleading (King *et al*. 2013; Armbruster 2017), mostly if pollen transfer and seed production are mainly carried out by a subset of the floral visitors. The magnitude of generalisation of the *M. arvensis* pollination system, quantified as the diversity of interactions, changed when quantified as the proportional contribution of each pollinator type to the production of seeds rather than as the proportion of visits made to the flowers, and this change went in opposite directions to each of the two plastic floral morphs. The pollination system of spring flowers was more specialised when considering the interaction at the level of seed production than when considered at the level of pollinator visitation rate. So, while the probability that two pollinators taken at random from spring flowers belonged to different species was 59 %, the probability that two seeds taken randomly from these same flowers were produced by two different pollinators fell to 49 %. In contrast, the pollination system of the summer flowers became more generalist when we took into account the seeds produced by each pollinator. This means that to get a more accurate picture of any pollination system, we suggest obtaining information on pollinator efficiency, not just their abundance on flowers. Traditionally neglected in pollination studies, knowledge of pollinator efficiency is crucial to characterise generalisation–specialisation gradients accurately, rigorously categorise pollination niches and correctly describe the architecture of plant-pollinator networks.

## Supporting Information

The following additional information is available in the online version of this article –

**Methods S1.** Complementary pollination-related traits of summer and spring morphs of *Moricandia arvensis*

**Table S1.** Pollinator functional groups. Brief description of the functional groups of the insects visiting the flowers of the studied species (Modified from Gómez *et al*. 2022)

**Table S2.** Total number of floral visits to the flowers of *Moricandia arvensis* used in the preference and effectiveness experiments by each pollinator functional group.

**Table S3.** Preference of pollinator functional groups for floral morphs. The table shows the values of the Jacob’s D index of preference (Jacobs 1974). Because this index is symmetric around zero, we only show the value for the electivity of spring floral morphs. We also show the number of insects of each functional group included in the experiments and the value of the goodness of fit. We show the outcomes of the experiments done both during spring and during summer.

**Table S4.** Outcome of the effectiveness analysis.

**Figure S1.** Results of the clustering analysis on the effectiveness of each pollinator functional group for each floral morph.

plae065_suppl_Supplementary_Material

plae065_suppl_Supplementary_Data

## Data Availability

All data used in this study has been included in the manuscript as Datasets S1-S4 and are hosted in https://digital.csic.es/handle/10261/361149.

## References

[CIT0001] Albrecht M, Schmid B, Hautier Y, Müller CB. 2012. Diverse pollinator communities enhance plant reproductive success. Proceedings Biological Sciences 279:4845–4852. 23034701 10.1098/rspb.2012.1621 PMC3497085

[CIT0002] Ali M, Saeed S, Sajjad A, Whittington A. 2011. In search of the best pollinators for canola (*Brassica napus* L.) production in Pakistan. Applied Entomology and Zoology 46:353–361.

[CIT0003] Anderson MJ. 2001. A new method for non–parametric multivariate analysis of variance. Austral Ecology 26:32–46.

[CIT0004] Armbruster WS. 2017. The specialization continuum in pollination systems: diversity of concepts and implications for ecology, evolution and conservation. Functional Ecology 31:88–100.

[CIT0005] Baddeley A, Rubak E, Turner R 2015. Spatial point patterns: Methodology and applications with R. Boca Ratón, USA: Chapman and Hall/CRC Press, London

[CIT0006] Barazani O, Erez T, Ogran A, Hanin N, Barzilai M, Dag A, Shafir S. 2019. Natural variation in flower color and scent in populations of *Eruca sativa* (Brassicaceae) affects pollination behavior of honey bees. Journal of Insect Science 19:1–9. 10.1093/jisesa/iez038 PMC6516435 31087084

[CIT0007] Barrett SCH. 2002. Sexual interference of the floral kind. Heredity 88:154–159. 11932774 10.1038/sj.hdy.6800020

[CIT0008] Bascompte J, Jordano P. 2014. Mutualistic networks. Princeton, USA: Princeton University Press.

[CIT0009] Baselga A, Orme CDL. 2012. betapart: an R package for the study of beta diversity. Methods in Ecology and Evolution 3:808–812.

[CIT0010] Bell G. 2010. Fluctuating selection: the perpetual renewal of adaptation in variable environments. Philosophical Transactions of the Royal Society of London, Series B: Biological Sciences 365:87–97. 20008388 10.1098/rstb.2009.0150 PMC2842698

[CIT0011] Bolnick DI, Yang LH, Fordyce JA, Davis JM, Svanbäck R. 2002. Measuring individual‐level resource specialization. Ecology 83:2936–2941.

[CIT0012] Bolnick DI, Svanbäck R, Fordyce JA, Yang LH, Davis JM, Hulsey CD, Forister ML. 2003. The ecology of individuals: incidence and implications of individual specialization. The American Naturalist 161:1–28. 10.1086/343878 12650459

[CIT0013] Borcard D, Gillet F, Legendre P. 2011. Numerical ecology with R. Vol. 2. New York, USA: Springer Jacobs.

[CIT0014] Bradshaw AD. 1965. Evolutionary significance of phenotypic plasticity in plants. Advances in Genetics 13:115–155.

[CIT0015] Brosi BJ. 2016. Pollinator specialization: from the individual to the community. The New Phytologist 210:1190–1194. 27038018 10.1111/nph.13951

[CIT0016] Calviño-Cancela M, Martín-Herrero J. 2009. Effectiveness of a varied assemblage of seed dispersers of a fleshy–fruited plant. Ecology 90:3503–3515. 20120817 10.1890/08-1629.1

[CIT0017] Cane JH, Payne JA. 1993. Regional, annual, and seasonal variation in pollinator guilds: intrinsic traits of bees (Hymenoptera: Apoidea) underlie their patterns of abundance at *Vaccinium ashei* (Ericaceae). Annals of the Entomological Society of America 86:577–588.

[CIT0018] Castro S, Loureiro J, Ferrero V, Silveira P, Navarro L. 2013. So many visitors and so few pollinators: variation in insect frequency and effectiveness governs the reproductive success of an endemic milkwort. Plant Ecology 214:1233–1245.

[CIT0019] Chao A, Gotelli NJ, Hsieh TC, Sander EL, Ma KH, Colwell RK, Ellison AM. 2014. Rarefaction and extrapolation with Hill numbers: a framework for sampling and estimation in species diversity studies. Ecological Monographs 84:45–67.

[CIT0020] Charrad M, Ghazzali N, Boiteau V, Niknafs A. 2014. NbClust: an R package for determining the relevant number of clusters in a data set. Journal of Statistical Software 61:1–36.

[CIT0022] Conner JK, Davis R, Rush S. 1995. The effect of wild radish floral morphology on pollination efficiency by four taxa of pollinators. Oecologia 104:234–245. 28307360 10.1007/BF00328588

[CIT0023] Cruden RW, McClain AM, Shrivastava GP. 1996. Pollination biology and breeding system of *Alliaria petiolata* (Brassicaceae). Bulletin of the Torrey Botanical Club 123:273–280.

[CIT0024] Dai C, Galloway LF. 2011. Do dichogamy and herkogamy reduce sexual interference in a self‐incompatible species? Functional Ecology 25:271–278.

[CIT0025] Dukas R, Shmida A. 1989. Correlation between the color, size and shape of Israeli crucifer flowers and relationships to pollinators. Oikos 54:281–286.

[CIT0026] Dupont YL, Padrón B, Olesen JM, Petanidou T. 2009. Spatio‐temporal variation in the structure of pollination networks. Oikos 118:1261–1269.

[CIT0027] Faegri K, van der Pijl L. 1979. Principles of pollination ecology, 3rd edn. London, UK: Pergamon.

[CIT0028] Fenster CB, Dudash MR. 2001. Spatiotemporal variation in the role of hummingbirds as pollinators of *Silene virginica*. Ecology 82:844–851.

[CIT0029] Fenster CB, Armbruster WS, Wilson P, Dudash MR, Thomson JD. 2004. Pollination syndromes and floral specialization. Annual Review of Ecology, Evolution, and Systematics 35:375–403.

[CIT0030] Fishbein M, Venable DL. 1996. Diversity and temporal change in the effective pollinators of *Asclepias tuberosa*. Ecology 77:1061–1073.

[CIT0031] Fisogni A, Rossi M, Sgolastra F, Bortolotti L, Bogo G, de Manincor N, Quaranta M, Galloni M. 2016. Seasonal and annual variations in the pollination efficiency of a pollinator community of *Dictamnus albus* L. Plant biology (Stuttgart, Germany) 18:445–454. 26573095 10.1111/plb.12417

[CIT0032] Földesi R, Howlett BG, Grass I, Batáry P. 2021. Larger pollinators deposit more pollen on stigmas across multiple plant species—a meta‐analysis. Journal of Applied Ecology 58:699–707.

[CIT0033] Fulkerson JR, Whittall JB, Carlson ML. 2012. Reproductive ecology and severe pollen limitation in the polychromic tundra plant, *Parrya nudicaulis* (Brassicaceae). PLoS One 7:e32790. 22427886 10.1371/journal.pone.0032790 PMC3299698

[CIT0034] Gómez JM. 2000. Effectiveness of ants as pollinators of *Lobularia maritima*: effects on main sequential fitness components of the host plant. Oecologia 122:90–97. 28307961 10.1007/PL00008840

[CIT0035] Gómez JM, Zamora R. 1999. Generalization vs. specialization in the pollination system of *Hormathophylla spinosa* (Cruciferae). Ecology 80:796–805.

[CIT0036] Gómez JM, Zamora R. 2006. Ecological factors that promote the evolution of generalization in pollination systems. In: Waser N, Ollerton J, eds. Plant–pollinator interactions: from specialization to generalization. Chicago, USA: The University of Chicago Press, 145–166.

[CIT0037] Gómez JM, Bosch J, Perfectti F, Fernández J, Abdelaziz M. 2007. Pollinator diversity affects plant reproduction and recruitment: the tradeoffs of generalization. Oecologia 153:597–605. 17576602 10.1007/s00442-007-0758-3

[CIT0038] Gómez JM, Bosch J, Perfectti F, Fernández JD, Abdelaziz M, Camacho JPM. 2008. Spatial variation in selection on corolla shape in a generalist plant is promoted by the preference patterns of its local pollinators. Proceedings Biological Sciences 275:2241–2249. 18544510 10.1098/rspb.2008.0512 PMC2603243

[CIT0039] Gómez JM, Muñoz-Pajares AJ, Abdelaziz M, Lorite J, Perfectti F. 2014. Evolution of pollination niches and floral divergence in the generalist plant *Erysimum mediohispanicum*. Annals of Botany 113:237–249. 23965614 10.1093/aob/mct186 PMC3890381

[CIT0040] Gómez JM, Perfectti F, Abdelaziz M, Lorite J, Muñoz‐Pajares AJ, Valverde J. 2015. Evolution of pollination niches in a generalist plant clade. New Phytologist 205:440–453. 25252267 10.1111/nph.13016

[CIT0041] Gómez JM, Torices R, Lorite J, Klingenberg CP, Perfectti F. 2016. The role of pollinators in the evolution of corolla shape variation, disparity and integration in a highly diversified plant family with a conserved floral bauplan. Annals of Botany 117:889–904. 26884512 10.1093/aob/mcv194 PMC4845802

[CIT0042] Gómez JM, Perfectti F, Armas C, Narbona E, González-Megías A, Navarro L, DeSoto L, Torices R. 2020. Within-individual phenotypic plasticity in flowers fosters pollination niche shift. Nature Communications 11:4019. 10.1038/s41467-020-17875-1 PMC7419554 32782255

[CIT0043] Gómez JM, González-Megías A, Narbona E, Navarro L, Perfectti F, Armas C. 2022a. Phenotypic plasticity guides *Moricandia arvensis* divergence and convergence across the Brassicaceae floral morphospace. The New Phytologist 233:1479–1493. 34657297 10.1111/nph.17807

[CIT0044] Gómez JM, Schupp EW, Jordano P. 2022b. The ecological and evolutionary significance of effectiveness landscapes in mutualistic interactions. Ecology Letters 25:264–277. 34971487 10.1111/ele.13939

[CIT0045] Gómez JM, González-Megías A, Armas C, Narbona E, Navarro L, Perfectti F. 2024. Selection maintains a nonadaptive floral polyphenism. Evolution Letters 8:610–621. 39100232 10.1093/evlett/qrae017 PMC11291621

[CIT0046] González-Castro A, Calviño-Cancela M, Nogales M. 2015. Comparing seed dispersal effectiveness by frugivores at the community level. Ecology 96:808–818. 26236876 10.1890/14-0655.1

[CIT0047] González-Megías A. 2016. Within‐and trans‐generational effects of herbivores and detritivores on plant performance and reproduction. Journal of Animal Ecology 85:283–290. 26433200 10.1111/1365-2656.12453

[CIT0048] Guitián J, Guitian P, Navarro L. 1996. Spatio–temporal variation in the interactions between *Cornus sanguinea* and its pollinators. Acta Oecologica 17:285–295.

[CIT0132] Herrera CM. 1987. Components of pollinator ‘quality’: comparative analysis of a diverse insect assemblage. *Oikos* 50:79–90.

[CIT0049] Herrera CM. 1988. Variation in mutualisms: the spatiotemporal mosaic of a pollinator assemblage. Biological Journal of the Linnean Society 35:95–125.

[CIT0050] Herrera CM. 1995. Microclimate and individual variation in pollinators: flowering plants are more than their flowers. Ecology 76:1516–1524.

[CIT0051] Herrera CM. 2000. Flower–to–seedling consequences of different pollination regimes in an insect–pollinated shrub. Ecology 81:15–29.

[CIT0052] Horvitz CC, Schemske DW. 1990. Spatiotemporal variation in insect mutualists of a neotropical herb. Ecology 71:1085–1097.

[CIT0053] Hsieh TC, Ma K, Chao A. 2016. iNEXT: an R package for rarefaction and extrapolation of species diversity (H ill numbers). *Methods in Ecology and Evolution* 7:1451–1456.

[CIT0054] Hurlbert SH. 1971. The nonconcept of species diversity: a critique and alternative parameters. Ecology 52:577–586. 28973811 10.2307/1934145

[CIT0130] Inouye DW, Gill DE, Dudash MR, Fenster CB. 1994. A model and lexicon for pollen fate. *American Journal of Botany* 81:1517–1530.

[CIT0055] Irwin RE, Bronstein JL, Manson JS, Richardson L. 2010. Nectar robbing: ecological and evolutionary perspectives. Annual Review of Ecology, Evolution, and Systematics 41:271–292.

[CIT0056] Ivey CT, Martinez P, Wyatt R. 2003. Variation in pollinator effectiveness in Swamp milkweed, *Asclepias incarnata* (Apocynaceae). American Journal of Botany 90:214–225. 21659111 10.3732/ajb.90.2.214

[CIT0057] Ivlev VS. 1961. Experimental ecology of the feeding of fishes. New Haven, Connecticut, USA: Yale University Press.

[CIT0058] Jacobs J. 1974. Quantitative measurement of food selection. Oecologia 14:413–417. 28308662 10.1007/BF00384581

[CIT0059] Jauker F, Wolters V. 2008. Hover flies are efficient pollinators of oilseed rape. Oecologia 156:819–823. 18438687 10.1007/s00442-008-1034-x

[CIT0060] Jauker F, Bondarenko B, Becker HC, Steffan‐Dewenter I. 2012. Pollination efficiency of wild bees and hoverflies provided to oilseed rape. Agricultural and Forest Entomology 14:81–87.

[CIT0061] Johnson SD. 2010. The pollination niche and its role in the diversification and maintenance of the southern African flora. Philosophical Transactions of the Royal Society of London, Series B: Biological Sciences 365:499–516. 20047876 10.1098/rstb.2009.0243 PMC2838267

[CIT0062] Kindt RCR. 2005. Tree diversity analysis. A manual and software for common statistical methods for ecological and biodiversity studies. Nairobi, Kenya: World Agroforestry Centre (ICRAF). ISBN 92–9059–179–X, https://www.cifor-icraf.org/publications/downloads/Publications/PDFS/b13695.pdf

[CIT0063] King C, Ballantyne G, Willmer PG. 2013. Why flower visitation is a poor proxy for pollination: measuring single‐visit pollen deposition, with implications for pollination networks and conservation. Methods in Ecology and Evolution 4:811–818.

[CIT0064] Küchmeister H, Shmida A, Gottsberger G. 1995. Phenology and pollination ecology of the desert plant *Moricandia nitens* (Brassicaceae) in the Negev, Israel. Advances in Geoecology 28:157–171.

[CIT0065] Lechowicz MJ. 1982. The sampling characteristics of electivity indices. Oecologia 52:22–30. 28310104 10.1007/BF00349007

[CIT0066] Li DF, Yan XC, Lin Y, Wang L, Wang Q. 2021. Do flowers removed of either nectar or pollen attract fewer bumblebee pollinators? An experimental test in *Impatiens oxyanthera*. AoB Plants 13:plab029. 34234935 10.1093/aobpla/plab029 PMC8255076

[CIT0067] Magurran AE. 2004. Measuring biological diversity. Oxford, UK: Blackwell Publishing.

[CIT0068] Maloof JE, Inouye DW. 2000. Are nectar robbers cheaters or mutualists? Ecology 81:2651–2661.

[CIT0069] Manetas Y, Petropoulou Y. 2000. Nectar amount, pollinator visit duration and pollination success in the Mediterranean shrub *Cistus creticus*. Annals of Botany 86:815–820.

[CIT0070] Matsuki Y, Tateno R, Shibata M, Isagi Y. 2008. Pollination efficiencies of flower‐visiting insects as determined by direct genetic analysis of pollen origin. American Journal of Botany 95:925–930. 21632415 10.3732/ajb.0800036

[CIT0071] McGuire AD, Armbruster WS. 1991. An experimental test for reproductive interactions between two sequentially blooming *Saxifraga* species (Saxifragaceae). American Journal of Botany 78:214–219.

[CIT0072] Méndez‐Vigo B, Gomaa NH, Alonso‐Blanco C, Picó XF. 2013. Among‐and within‐population variation in flowering time of Iberian *Arabidopsis thaliana* estimated in field and glasshouse conditions. New Phytologist 197:1332–1343. 23252608 10.1111/nph.12082

[CIT0073] Moreira-Hernández JI, Muchhala N. 2019. Importance of pollinator–mediated interspecific pollen transfer for angiosperm evolution. Annual Review of Ecology, Evolution, and Systematics 50:191–217.

[CIT0074] Motten AF, Campbell DR, Alexander DE, Miller HL. 1981. Pollination effectiveness of specialist and generalist visitors to a North Carolina population of *Claytonia virginica*. Ecology 62:1278–1287.

[CIT0075] Murren CJ, Auld JR, Callahan H, Ghalambor CK, Handelsman CA, Heskel MA, Kingsolver JG, Maclean HJ, Masel J, Maughan H, et al 2015. Constraints on the evolution of phenotypic plasticity: limits and costs of phenotype and plasticity. Heredity 115:293–301. 25690179 10.1038/hdy.2015.8 PMC4815460

[CIT0129] Ne’eman G, Jürgens A, Newstrom-Lloyd L, Potts SG, Dafni A. 2010. A framework for comparing pollinator performance: effectiveness and efficiency. *Biological Reviews* 85:435–451 20015317 10.1111/j.1469-185X.2009.00108.x

[CIT0076] Neal PR, Dafni A, Giurfa M. 1998. Floral symmetry and its role in plant–pollinator systems: terminology, distribution, and hypotheses. Annual Review of Ecology, Evolution, and Systematics 29:345–373.

[CIT0077] Nikolov LA. 2019. Brassicaceae flowers: diversity amid uniformity. Journal of Experimental Botany 70:2623–2635. 30824938 10.1093/jxb/erz079

[CIT0078] Nocentini D, Pacini E, Guarnieri M, Martelli D, Nepi M. 2013. Intrapopulation heterogeneity in floral nectar attributes and foraging insects of an ecotonal Mediterranean species. Plant Ecology 214:799–809.

[CIT0079] Oksanen J, Simpson G, Blanchet F, Kindt R, Legendre P, Minchin P, O’Hara R, Solymos P, Stevens M, Szoecs E, et al 2022. vegan: community ecology package. R package version 2.6–4, https://github.com/vegandevs/vegan

[CIT0080] Olesen JM, Eskildsen LI, Venkatasamy S. 2002. Invasion of pollination networks on oceanic islands: importance of invader complexes and endemic super generalists. Diversity and Distributions 8:181–192.

[CIT0081] Ollerton J, Killick A, Lamborn E, Watts S, Whiston M. 2007. Multiple meanings and modes: on the many ways to be a generalist flower. Taxon 56:717–728.

[CIT0135] Olsen KM. 1997. Pollination effectiveness and pollinator importance in a population of Heterotheca subaxillaris (Asteraceae). *Oecologia* 109:114–121. 10.1007/PL00008811 28307601

[CIT0082] Page ML, Nicholson CC, Brennan RM, Britzman AT, Greer J, Hemberger J, Kahl H, Müller U, Peng Y, Rosenberger NM, et al 2021. A meta‐analysis of single visit pollination effectiveness comparing honeybees and other floral visitors. American Journal of Botany 108:2196–2207. 34622948 10.1002/ajb2.1764

[CIT0083] Palacio FX. 2019. Seed dispersal effectiveness by frugivorous birds: identifying functional equivalent species in bird assemblages. Avian Biology Research 12:103–108.

[CIT0084] Patiny S. 2012. Evolution of plant–pollinator relationships. Cambridge, UK: Cambridge University Press.

[CIT0085] Perfectti F, Gómez JM, González-Megías A, Abdelaziz M, Lorite J. 2017. Molecular phylogeny and evolutionary history of *Moricandia* DC (Brassicaceae). PeerJ 5:e3964. 29093999 10.7717/peerj.3964 PMC5661452

[CIT0086] Pesenko YA, Radchenko VG, Kaygorodova MS. 1980. Ecology of pollination in *Strigosella grandiflora* and *Erysimum badghysi* (Brassicaceae) by bees (Hymenoptera, Apoidea) in Badkhyz: Estimation of the pressure of competitive relationships. Entomological Review 59:58–74.

[CIT0087] Phillips BB, Williams A, Osborne JL, Shaw RF. 2018. Shared traits make flies and bees effective pollinators of oilseed rape (*Brassica napus* L.). Basic and Applied Ecology 32:66–76.

[CIT0088] Phillips RD, Peakall R, van der Niet T, Johnson SD. 2020. Niche perspectives on plant–pollinator interactions. Trends in Plant Science 25:779–793. 32386827 10.1016/j.tplants.2020.03.009

[CIT0089] Pleasants JM. 1983. Nectar production patterns in *Ipomopsis aggregata* (Polemoniaceae). American Journal of Botany 70:1468–1475.

[CIT0090] Prado SG, Collazo JA, Marand MH, Irwin RE. 2021. The influence of floral resources and microclimate on pollinator visitation in an agro–ecosystem. Agriculture, Ecosystems and Environment 307:107196.

[CIT0091] Preston RE. 1994. Pollination biology of *Streptanthus tortuosus* (Brassicaceae). Madroño 41:138–147.

[CIT0092] Price MV, Waser NM, Irwin RE, Campbell DR, Brody AK. 2005. Temporal and spatial variation in pollination of a montane herb: a seven‐year study. Ecology 86:2106–2116.

[CIT0134] Primack RB, Silander JA. 1975. Measuring the relative importance of different pollinators to plants. *Nature* 255:143–144.

[CIT0094] Rader R, Howlett BG, Cunningham SA, Westcott DA, Edwards W. 2012. Spatial and temporal variation in pollinator effectiveness: do unmanaged insects provide consistent pollination services to mass flowering crops? Journal of Applied Ecology 49:126–134.

[CIT0095] Rader R, Edwards W, Westcott DA, Cunningham SA, Howlett BG. 2013. Diurnal effectiveness of pollination by bees and flies in agricultural *Brassica rapa*: implications for ecosystem resilience. Basic and Applied Ecology 14:20–27.

[CIT0096] Ramírez-Valiente JA, Valladares F, Delgado A, Nicotra AB, Aranda I. 2015. Understanding the importance of intrapopulation functional variability and phenotypic plasticity in *Quercus suber*. Tree Genetics and Genomes 11:1–11.

[CIT0097] Real L, Rathcke BJ. 1988. Patterns of individual variability in floral resources. Ecology 69:728–735.

[CIT0098] Richardson J. 2020. selectapref: Analysis of field and laboratory foraging. R Manual. URL https://github.com/cran/selectapref

[CIT0099] Robertson IC, Klemash D. 2003. Insect–mediated pollination in slickspot peppergrass, *Lepidium papilliferum* L. (Brassicaceae), and its implications for population viability. Western North American Naturalist 63:333–342.

[CIT0100] Robertson IC, Leavitt H. 2011. Relative contributions to seed production by floral visitors of slickspot peppergrass, *Lepidium papilliferum* (Brassicaceae). Arthropod-Plant Interactions 5:379–389.

[CIT0101] Roy S, Gayen AK, Mitra B, Duttagupta A. 2014. Diversity, foraging activities of the insect visitors of Mustard (*Brassica juncea* Linnaeus) and their role in pollination in West Bengal. Journal of Zoology Studies 1:7–12.

[CIT0133] Sahli HF, Conner JK. 2006. Characterizing ecological generalization in plant-pollination systems. *Oecologia* 148:365–372. 16514533 10.1007/s00442-006-0396-1

[CIT0102] Sahli HF, Conner JK. 2007. Visitation, effectiveness, and efficiency of 15 genera of visitors to wild radish, *Raphanus raphanistrum* (Brassicaceae). American Journal of Botany 94:203–209. 21642222 10.3732/ajb.94.2.203

[CIT0103] Salisbury EJ. 1965. The reproduction of *Cardamine pratensis* L. and *Cardamine palustris* Peterman particularly in relation to their specialized foliar vivipary, and its deflexion of the constraints of natural selection. Proceedings of the Royal Society of London. Series B 163:321–342.

[CIT0104] Scheiner SM. 2013. The genetics of phenotypic plasticity. XII. Temporal and spatial heterogeneity. Ecology and Evolution 3:4596–4609. 24340198 10.1002/ece3.792 PMC3856757

[CIT0105] Schemske DW, Horvitz CC. 1989. Temporal variation in selection on a floral character. Evolution 43:461–465. 28568553 10.1111/j.1558-5646.1989.tb04240.x

[CIT0106] Schlichting CD, Pigliucci M. 1998. Phenotypic evolution, a reaction norm perspective. Sunderland, USA: Sinauer. Associates.

[CIT0107] Schupp EW, Jordano P, Gómez JM. 2010. Seed dispersal effectiveness revisited: a conceptual review. The New Phytologist 188:333–353. 20673283 10.1111/j.1469-8137.2010.03402.x

[CIT0108] Schupp EW, Jordano P, Gómez JM. 2017. A general framework for effectiveness concepts in mutualisms. Ecology Letters 20:577–590. 28349589 10.1111/ele.12764

[CIT0109] Sentil A, Reverté S, Lhomme P, Bencharki Y, Rasmont P, Christmann S, Michez D. 2022. Wild vegetation and ‘farming with alternative pollinators’ approach support pollinator diversity in farmland. Journal of Applied Entomology 146:1155–1168.

[CIT0110] Shakeel M, Ali H, Ahmad S, Said F, Khan KA, Bashir MA, Anjum SI, Islam W, Ghramh HA, Ansari MJ, et al 2019. Insect pollinators diversity and abundance in *Eruca sativa* Mill. (Arugula) and *Brassica rapa* L. (Field mustard) crops. Saudi Journal of Biological Sciences 26:1704–1709. 31762647 10.1016/j.sjbs.2018.08.012 PMC6864147

[CIT0111] Siepielski AM, DiBattista JD, Carlson SM. 2009. It’s about time: the temporal dynamics of phenotypic selection in the wild. Ecology Letters 12:1261–1276. 19740111 10.1111/j.1461-0248.2009.01381.x

[CIT0112] Streher NS, Budinsky T, Halabi K, Mayrose I, Ashman TL. 2024. The effect of polyploidy and mating system on floral size and the pollination niche in Brassicaceae. International Journal of Plant Sciences 185:89–99.

[CIT0113] Strzalkowska–Abramek M, Tymoszuk K, Jachula J, Bozek M. 2016. Nectar and pollen production in *Arabis procurrens* Waldst. and Kit. and *Iberis sempervirens* L.(Brassicaceae). Acta Agrobotanica 69:1–10.

[CIT0114] Szenteczki MA, Godschalx AL, Galmán A, Espíndola A, Gibernau M, Alvarez N, Fishbein Rasmann S. 2021. Spatial and temporal heterogeneity in pollinator communities maintain within‐species floral odour variation. Oikos 130:1487–1499.

[CIT0115] Thompson JN. 2005. The geographic mosaic of coevolution. Chicago, USA: The University of Chicago Press.

[CIT0116] Traveset A, Sáez E. 1997. Pollination of *Euphorbia dendroides* by lizards and insects: spatio–temporal variation in patterns of flower visitation. Oecologia 111:241–248. 28308000 10.1007/PL00008816

[CIT0117] Valverde J, Gómez JM, Perfectti F. 2016. The temporal dimension in individual‐based plant pollination networks. Oikos 125:468–479.

[CIT0118] Valverde J, Perfectti F, Gómez JM. 2019. Pollination effectiveness in a generalist plant: adding the genetic component. The New Phytologist 223:354–365. 30761538 10.1111/nph.15743

[CIT0119] Waites AR, Agren J. 2006. Stigma receptivity and effects of prior self–pollination on seed set in tristylous *Lythrum salicaria* (Lythraceae). American Journal of Botany 93:142–147.

[CIT0120] Waser NM, Ollerton J. 2006. Plant–pollinator interactions: from specialization to generalization. Chicago, USA: The University of Chicago Press.

[CIT0121] Waser NM, Chittka L, Price MV, Williams NM, Ollerton J. 1996. Generalization in pollination systems, and why it matters. Ecology 77:1043–1060.

[CIT0122] Wiegand T, Moloney KA. 2014. Handbook of spatial point–pattern analysis in ecology. Boca Ratón, USA: CRC Press.

[CIT0123] Willmer P 2011. Pollination and floral ecology. Princeton, USA: Princeton University Press.

[CIT0124] Wu Y, Li QJ. 2017. Phenotypic selection on flowering phenology and pollination efficiency traits between *Primula* populations with different pollinator assemblages. Ecology and Evolution 7:7599–7608. 29043017 10.1002/ece3.3258 PMC5632619

[CIT0125] Yadav S, Jat MK, Yadav SS, Kumar H. 2022. Diversity, abundance and foraging behaviour of pollinators in early sown rapeseed–mustard genotypes. Journal of Agriculture and Ecology 14:104–112.

[CIT0126] Zamora R. 2000. Functional equivalence in plant‐animal interactions: ecological and evolutionary consequences. Oikos 88:442–447.

[CIT0127] Zych M, Junker RR, Nepi M, Stpiczyńska M, Stolarska B, Roguz K. 2019. Spatiotemporal variation in the pollination systems of a supergeneralist plant: is *Angelica sylvestris* (Apiaceae) locally adapted to its most effective pollinators? Annals of Botany 123:415–428. 30059963 10.1093/aob/mcy140 PMC6344219

