# Supplementary material for: Pollination effectiveness affects the level of generalisation of a plant species with phenotypically plastic flowers
Source: AoB Plants. 2025 Jan 10;17(3):plae065. doi: 10.1093/aobpla/plae065 (PMC12190806; doi:10.1093/aobpla/plae065)

Supplementary Methods

Method S1. Complementary pollination-related traits of summer and spring morphs of *Moricandia arvensis*

To complete the data set presented in Table 1, in this work we have collected information on four new floral traits related to the relative position of floral organs, herkogamy, and sexual organ exsertion. Because the flowers of this species present their stamens in two whorls, we calculated two measures of herkogamy as the difference between the height of the stigma and the insertion point of the anthers in each of the staminal whorls. We also calculated the degree of stigma exertion as the difference between the height of stigma and the upper edge of the corolla tube, and the exertion of the stamens as the difference between the height of the stamens of upper whorl and the upper edge of the corolla tube. These variables were expressed in mm and measured using a digital caliper with ± 0.1 mm of error. Finally, we collected information on three reward traits: production of nectar, concentration of nectar, and sugar quantity per flower. The production of nectar for each floral morph, expressed in **μ**L per flower, was evaluated in 2022 in the field both during spring and summer. For each floral morph, five unvisited flowers from six plants were selected in the field and their nectar volume measured using capillary mi-cropipettes. The sugar concentration of this nectar, expressed as the percentage of sugar by weight, was deter-mined using a portable refractometer Bellingham and Stanley designed explicitly for small volumes of nectar. The amount of sugar produced by each flower, expressed in mg, was estimated according to Prŷs-Jones and Corbet (1991).

Supplementary Tables

Table S1. Pollinator functional groups. Brief description of the functional groups of the insects visiting the flowers of the studied species (Modified from Gómez *et al.* 2022).

Code	Functional Group	Body length	Resource	Behavioural notes	Type of visits	Order	Examples
1	Long-tongued large bees	10-15 mm	Nectar + Pollen	Partially introducing the head in the flower	Legitimate	Hymenoptera	Anthophoridae
2	Long-tongued medium-sized bees	< 10 mm	Nectar + Pollen	Partially introducing the head in the flower	Legitimate	Hymenoptera	Anthophoridae
3	Short-tongued large bees	> 10 mm	Pollen + Nectar	Introducing the whole head in the flower	Legitimate	Hymenoptera	Halictidae, Megachilidae, Colletidae Andrenidae
4	Short-tongued medium-sized bees	5 – 10 mm	Pollen + Nectar	Introducing the whole head in the flower	Legitimate	Hymenoptera	Halictidae, Colletidae, Andrenidae, Apidae Xylocopinae, Apidae Nomidinae
5	Short-tongued small bees	2 – 5 mm	Pollen + Nectar	They access the nectar legitimately or from between the sepals	Illegitimate + Legitimate	Hymenoptera	Halictidae, Colletidae, Andrenidae, Apidae Xylocopinae, Apidae Nomidinae
6	Short-tongued extra-small bees	< 2 mm	Nectar + Pollen	They access the nectar legitimately or from between the sepals	Legitimate + Illegitimate	Hymenoptera	Halictidae, Colletidae
7	Honeybees	6-12 mm	Nectar + Pollen	Introducing the whole head in the flower	Legitimate	Hymenoptera	Apidae (*Apis* spp.)
8	Pollen wasps	Variable	Pollen	Partially introducing the head in the flower	Legitimate	Hymenoptera	Massarinae
9	Large nectar-feeding wasps	> 7mm	Nectar	Partially introducing the head in the flower	Legitimate	Hymenoptera	Vespidae
10	Small nectar-feeding wasps	Usually < 3mm	Nectar	Mostly sipping nectar from between sepals	Illegitimate + Legitimate	Hymenoptera	Chalcidoidea, Ichneumonoidea
11	Large ants	> 2 mm	Nectar	They can introduce the whole body in the flower to reach the nectar	Legitimate + Illegitimate	Hymenoptera	Formicidae
12	Small ants	< 2 mm	Nectar	Mostly sipping nectar from between sepals	Illegitimate + Legitimate	Hymenoptera	Formicidae
13	Hovering beeflies	Variable	Nectar + Pollen	Hovering while collecting nectar from the inside of floral tubes and also some pollen with their long proboscides	Legitimate	Diptera	Bombyliidae (*Bombylius*)

14	Non-hovering beeflies	Variable	Nectar	Collecting nectar with long buccal apparatus without hovering;	Legitimate	Diptera	Bombyliidae, Tachinidae, Nemestrinidae,
15	Long-tongued flies	< 5 mm	Nectar	Sipping nectar	Illegitimate + Legitimate	Diptera	Bibionidae, Empididae
16	Large hoverflies	>5 mm	Pollen	Collect pollen without entering the flower	Legitimate	Diptera	Syrphidae (Eristalini)
17	Small hoverflies	< 5 mm	Pollen + Nectar	Collect pollen without entering the flower and sometimes sip nectar from between the sepals	Legitimate + Illegitimate	Diptera	Syrphidae
18	Large flies	>5 mm	Nectar + Pollen	Collect pollen without entering the flower and nectar	Legitimate + Illegitimate	Diptera	Muscidae, Calliphoridae, Tabanidae, Scatophagidae, Anthomyiidae
19	Small flies	< 5 mm	Nectar + Pollen	Mostly sipping nectar	Illegitimate + Legitimate	Diptera	Muscidae, Anthomyiidae, Micetophyliidae, Drosophilidae, Stratiomyidae
20	Large butterflies	≥ 20 mm	Nectar	Feeding on nectar both from inside the flower and between the sepals	Legitimate	Lepidoptera	Nymphalidae, Papilionidae, Pieridae
21	Small butterflies	< 20 mm	Nectar	Feeding on nectar both from inside the flower and between the sepals	Legitimate	Lepidoptera	Lycaenidae, Pieridae, Hesperidae
22	Hawkmoths	> 7 mm	Nectar	Hovering to sip nectar	Legitimate	Lepidoptera	Sphingidae
23	Small moths	< 3mm	Nectar	Sipping nectar without entering the flower	Illegitimate + Legitimate	Lepidoptera	Adelidae, Plutellidae
24	Large beetles	> 7 mm	Mostly Pollen	Consuming not only pollen, also anthers, petals, and other floral parts	Legitimate + Illegitimate	Coleoptera	Cetonidae, Lagridae, Mylabridae, Allecuninae
25	Small beetles	< 7 mm	Pollen + Nectar	Consuming pollen during legitimate visits also robbing nectar from the bottom part of the flowers	Legitimate + Illegitimate	Coleoptera	Melyridae (Malachidae, Dasytidae), Cleridae, Oedemeridae, Elateridae, Bruchidae, Buprestidae, Chrysomelidae
26	Small diving beetles	<3 mm	Nectar + Pollen	Entering completely into the flower, crawling down the corolla for nectar	Legitimate	Coleoptera	Nitidulidae, Dermestidae, Phalacridae
27	Aphids	< 2 mm	Nectar	Mostly winged individuals	Legitimate	Hemiptera	Aphidoidea
28	Bugs	variable	Nectar	Sipping nectar without entering the flower. Also acting as sapsuckers in vegetative tissues	Legitimate + Illegitimate	Hemiptera	Lygaeidae, Pentatomidae
29	Thrips	< 3 mm	Pollen	Feeding from inside the flowers	Legitimate	Thysanoptera	

Table S2. Total number of floral visits to the flowers of *Moricandia arvensis* used in the preference and effectiveness experiments by each pollinator functional group.

	Preference Experiment				Effectiveness Experiment	
**Season**	Spring		Summer			
**Floral Morph**	Spring	Summer	Spring	Summer	Spring	Summer
**Hymenoptera**						
Long-tongued large bees	832	43	280	195	105	37
Short-tongued large bees	1	1	82	61	46	21
Short-tongued medium-sized bees	8	4	36	31	98	60
Short-tongued small bees	28	4	1	0	88	69
Short-tongued extra small bees	2	0	0	1	4	7
Large nectaring wasps	0	0	0	0		
Small nectaring wasps	9	0	2	6		
Large ants	0	1	0	0		
**Diptera**						
Hovering beeflies	31	4	38	18	92	39
Non-hovering beeflies					11	
Long-tongued flies	18	15	0	0		
Large hoverflies	0	0	0	2	16	19
Small hoverflies	36	20	2	2	11	5
Large flies	0	31	0	0		3
Small flies	1	8	0	0	1	2
**Lepidoptera**						
Large butterflies	196	114	279	339	42	64
Small butterflies	0	0	69	24	5	3
Hawkmoths	9	0	0	0		
**Coleoptera**						
Small beetles					4	0

Table S3. Preference of pollinator functional groups for floral morphs. The table shows the values of the Jacob's D index of preference (Jacobs 1974). Because this index is symmetric around zero, we only show the value for the electivity of spring floral morphs. We also show the number of insects of each functional group included in the experiments and the value of the goodness of fit. We show the outcomes of the experiments done both during spring and during summer.

	**SPRING**				**SUMMER**			
**Functional Group**	**Number of insects**	**Preference for spring floral morph**	**Chi square**	**P value**	**Number of insects**	**Preference for spring floral morph**	**Chi square**	**P value**
Long-tongued large bees	875	0.82	339.17	0.000	475	0.13	7.30	0.007
Short-tongued large bees	2	-0.31	0.21	0.645	143	0.09	1.21	0.272
Short-tongued medium-sized bees	12	0.03	0.01	0.931	67	0.02	0.03	0.874
Short-tongued small bees	32	0.57	6.86	0.009	1	1.00	0.90	0.344
Short-tongued extra-small bees	2	1.00	1.05	0.305	1	-1.00	1.12	0.291
Small nectar-feeding wasps	9	1.00	4.74	0.029	8	-0.54	2.47	0.116
Large ants	1	-1.00	1.90	0.168				
Large beeflies	35	0.61	8.25	0.004	56	0.31	5.12	0.024
Long-tongued flies	33	-0.23	1.75	0.186				
Large hoverflies					2	-1.00	2.23	0.135
Small hoverflies	56	-0.03	0.04	0.850	4	-0.06	0.01	0.912
Large flies	31	-1.00	58.82	0.000				
Small flies	9	-0.88	11.77	0.001				
Large butterflies	310	-0.05	0.70	0.402	618	-0.15	14.38	0.000
Small butterflies					93	0.44	17.14	0.000
Hawkmoths	9	1.00	4.74	0.029				

Table S4. Outcome of the effectiveness analysis.

FG	Ovules per flower	Fruit per flower	Seeds per fruit	Seeds per visit (QLC)	Visits per plant hr^{-1} (QTC)	Seeds per plant hr^{-1} (Effectiveness)	Proportional Effectiveness (%)
**Spring floral morph**							
Long-tongued large bees	52.5	0.63	26.2	16.5	50.53	831.7	77.99
Short-tongued large bees	64.3	0.80	25.0	20.1	1.57	31.6	2.96
Short-tongued medium-sized bees	57.0	0.56	26.2	14.7	1.57	23.1	2.17
Short-tongued small bees	44.7	0.40	13.4	5.3	7.60	40.4	3.79
Short-tongued extra small bees	43.7	0.75	23.7	17.8	0.37	6.5	0.61
Hovering beeflies	54.9	0.71	34.3	24.2	4.90	118.7	11.13
Non-hovering beeflies	45.6	0.82	35.2	28.8	0.04	1.1	0.00
Large hoverflies	4.0	0.06	0.0	0.0	0.04	0.0	0.00
Small hoverflies	44.0	0.09	35.0	3.2	0.07	0.2	0.02
Small flies	NA	0.00	NA	0.0	0.04	0.0	0.00
Large butterflies	60.1	0.48	26.2	12.5	1.13	14.1	1.32
Small butterflies	35.0	0.20	20.0	4.0	0.04	0.1	0.01
Small beetles	45.0	0.25	0.0	0.0	0.58	0.0	0.00
**Summer floral morph**							
Long-tongued large bees	45.6	0.60	27.6	16.4	11.42	187.6	19.21
Short-tongued large bees	45.0	0.38	25.4	9.7	24.50	237.4	24.31
Short-tongued medium-sized bees	41.5	0.52	20.5	10.6	5.16	54.5	5.58
Short-tongued small bees	43.7	0.33	13.4	4.5	57.11	255.1	26.12
Short-tongued extra small bees	48.3	0.43	33.3	14.3	11.79	168.4	17.24
Hovering beeflies	49.6	0.49	31.4	15.3	0.18	2.8	0.29
Large hoverflies	40.5	0.11	5.5	0.6	0.18	0.1	0.01
Small hoverflies	NA	0.00	NA	0.0	5.90	0.0	0.00
Large flies	36.0	0.33	4.0	1.3	0.92	1.2	0.12
Small flies	NA	0.00	NA	0.0	0.37	0.0	0.00
Large butterflies	43.2	0.562	24.9	14.0	4.97	69.6	7.13
Small butterflies	20.0	0.333	0.0	0.0	0.37	0.0	0.00
Small beetles	45.0	0.250	0.0	0.0	6.82	0.0	0.00

Figure S1. Results of the clustering analysis on the effectiveness of each pollinator functional group for each floral morph.

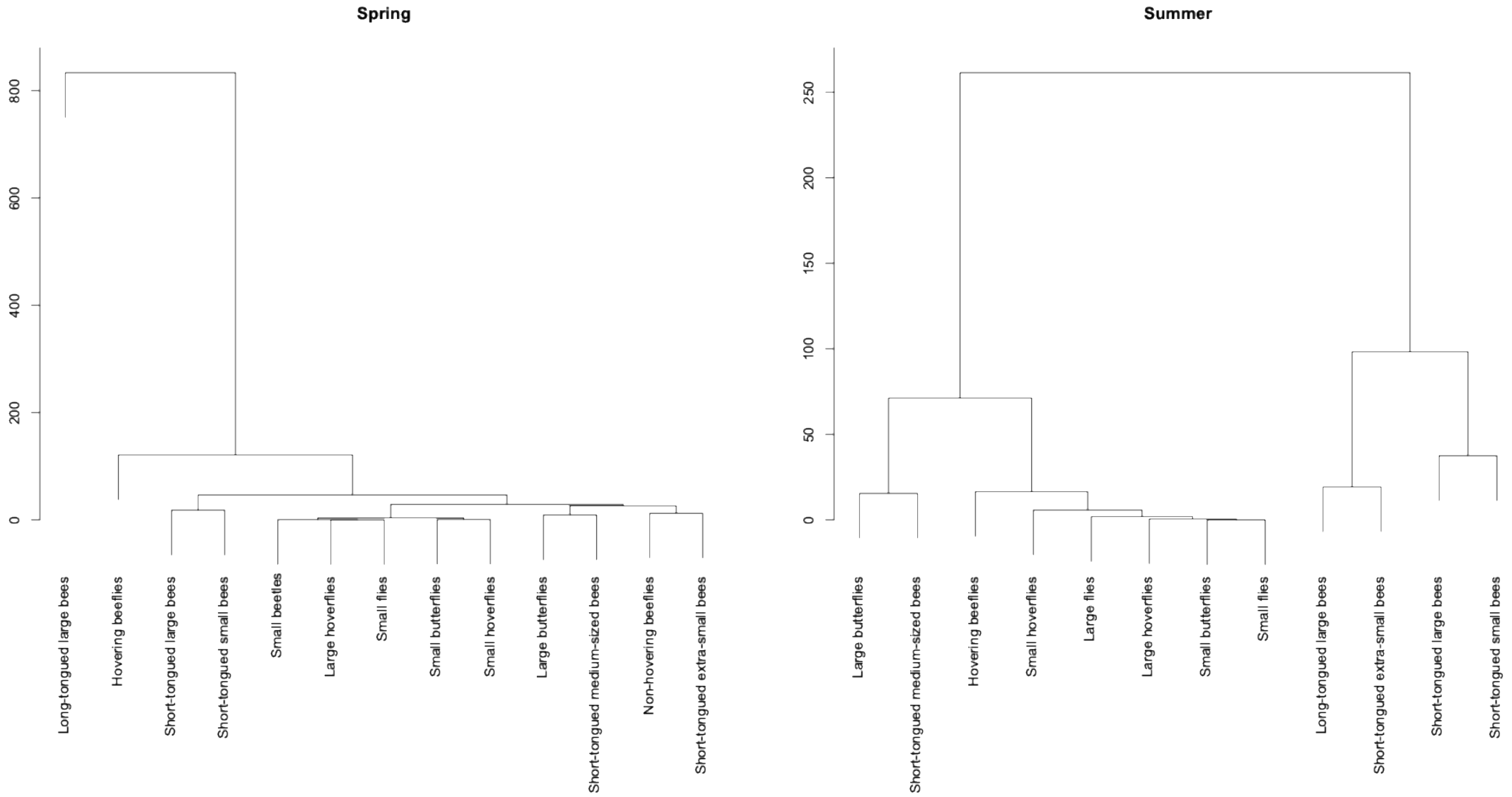

Supplement: plae065_suppl_Supplementary_Material [file plae065_suppl_supplementary_material.pdf]
